# Clinical validation and study of stem cell transplantation in treatment of vitiligo

**DOI:** 10.1007/s00403-023-02692-5

**Published:** 2023-09-07

**Authors:** Jingwei Liu, Shiyu Liu, Min Guo, Miao Yong, Zhiqi Hu

**Affiliations:** 1 Nanhai Renshu International Skin Hospital (Hainan) Co., Ltd., Haidian Sandong Road, Haikou, 570208 China; 2 Affiliated Hospital of Southern Medical University, Guangdong, 050100 China

**Keywords:** PCT, Vitiligo, Outer root sheath, Melanocyte stem cell, Melanocyte processing plant

As a clinical refractory disease, vitiligo has a significant impact on the physical and mental health of patients, threatening the state of their marriage, social interactions, and employment. As the pathogenesis of vitiligo remains unknown, the ineffective rate of various treatments for vitiligo patients has reached 50% [1]. Therefore, vitiligo has always been regarded as a chronic disease in dermatology. The new method for treating vitiligo invented by our team has been granted patents by the China Patent (Invention Patent) [2] (Technical Method for Treating Leucoderma Based on Hair Follicle Melanocyte Stem Cell Transplantation, Patent No.: ZL201910769979.1) and by the Patent Cooperation Treaty (PCT) [3] (Patent No.: PCT/CN2021/072340). “Technical method of hair follicle melanocytes stem cell transplantation for the treatment of vitiligo”, Patent number: ZL201910769979.1; also obtained PCT patent [4] patent number: PCT/CN2021/072340. To verify its efficacy, 50 patients with vitiligo who were treated from June 2021 to March 2022 and had a disease duration of 1–20 years and no response to various therapies were selected as the research objects. According to the homologous pairing experiment design, the PCT patented technology was clinically validated. Fifty patients in the treatment group were treated with PCT patent technology combined with 308 excimer laser once a week, and 50 patients in the control group were treated with 308 excimer laser only once a week. In the treatment group, 46 cases were cured and 4 cases were not cured (2 of them had hypothyroidism and took Euthyrox for a long time, and 2 were fingertip patients over 50 years). In the control group, 50 cases were not cured. The cure rate statistics showed that the total cure rate of the treatment group was 92%, and the cure rate of the control group was 0. By Chi-square test *χ*^2^ = 46 (*p* < 0.01), the PCT patent technology was significantly effective than the control group, and the difference in cure rate was statistically significant.

We have obtained McSCs in a functional state using a PCT-protected technical method and implanted them and melanoblasts to an area under the epidermis. Continuously activated by a 308-nm excimer laser in vitro, McSCs in the ORS were transformed into mature MCs and migrated along the ORS to multiple hair follicle orifices in the vitiligo area or sebaceous gland openings in the hairless area to achieve central-type repigmentation with no color difference. McSC transplantation addresses the issue of MC sources for patients with vitiligo [5] and provides a new solution for its treatment. With a cure rate of 92%, this method brings new hope for recovery to 70 million patients with vitiligo worldwide (Tables 1, 2).

**Table 1:** Treatment of vitiligo patients with PCT patent law and control group (*n*, %)

Statistical analysis			
Treatment condition	Surgical treatment group (*n* = 50)	Control group (*n* = 50)	Total
Cured			
Yes	46 (92)	0 (0)	46 (46)
No	4 (8)	50 (100)	54 (54)
Cutaneous pruritus			
Yes	1 (2.04)	0 (0)	1 (1)
No	49 (97.96)	50 (100)	99 (99)
Epifolliculitis			
Yes	4 (8)	0 (0)	4 (4)
No	46 (92)	50 (100)	96 (96)

**Table 2:** Comparison of cure of vitiligo patients with PCT patent method and control group (*n*)

Surgical treatment group	Control group		Total	*χ*^2^	*p*
	Cured	Uncured			
Cured	0	46	46	46	< 0.001
Uncured	0	4	4		

By Chi-square test *χ*^2^ = 46 (*p* < 0.01), the curative effect of PCT patent technology was significantly higher than that of the control group, and the difference in cure rate was statistically significant

**Fig. 1:**
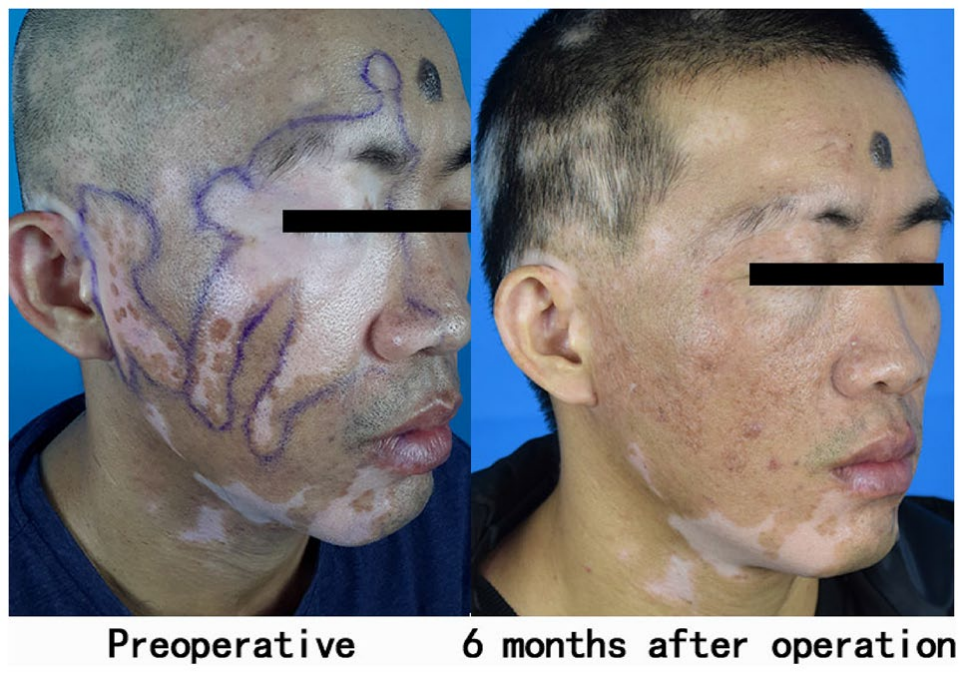
First operation

**Fig. 2:**
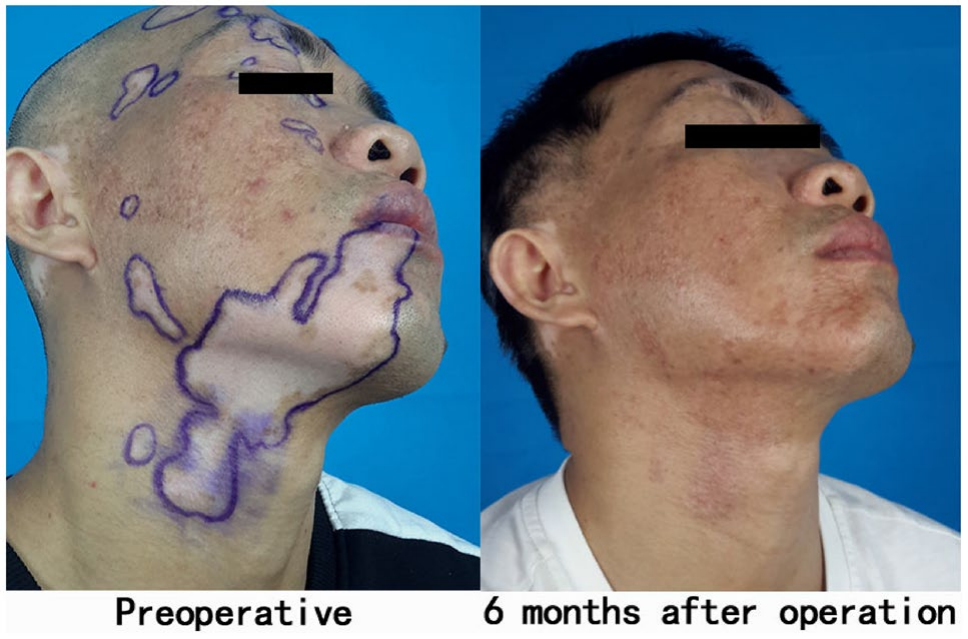
Second operation

A 35-year-old male patient presented with multiple depigmented leukoplakia on the right side of the face for 17 years (Figs. 1, 2).

### Supplementary Information

Below is the link to the electronic supplementary material.

Supplementary file1 (PDF 2373 KB)

Supplementary file2 (PDF 51673 KB)

Supplementary file3 (PDF 855 KB)

Supplementary file4 (PDF 2109 KB)

Supplementary file5 (PDF 1563 KB)
