# Supplementary material for: Clinical validation and study of stem cell transplantation in treatment of vitiligo
Source: Arch Dermatol Res. 2023 Sep 7;315(10):2983–4. doi: 10.1007/s00403-023-02692-5 (PMC10615963; doi:10.1007/s00403-023-02692-5)
Supplement: Supplementary file 1 — Supplementary file1 (PDF 2373 KB) [file 403_2023_2692_MOESM1_ESM.pdf]

肖像权使用协议书

甲方（肖像使用人）：海口仁术皮肤科门诊部有限公司

地址：海口市美兰区海甸三东路京海花园3铺3楼

联系人:刘景卫

电话：0898-66266732

电子邮件：renshupifu@163.com

乙方（肖像权人）：郭小鹏 身份证号：460022199010060715

住址：文昌市重兴镇宝树村

联系电话：15120608372

电子邮箱：

甲乙双方就乙方自愿授权甲方使用本人肖像，根据《中华人民共和国广告法》、《中华人民共和国民法典》的有关规定，为明确双方的权利义务关系，经平等、自愿、协商一致，达成如下，以资共同遵照执行。

一、一般约定

1、乙方为本协议中的肖像权人，自愿将个人的肖像权（含照片、视频、病患部位等全部影、音视频资料、作品及组合，以下统称为“肖像”）许可甲方作为商业或非商业用途使用。

2、甲方使用乙方肖像包括但不限于用于甲方案例展示、教学、网站、网络、微信等媒体；各种户外广告、宣传册、报纸期刊等画面；广告片、宣传片或其他用于甲方对内交流及对外营销宣传推广等所需要内

容。

3、使用地域范围包括国内和国外。

4、乙方许可甲方进行无限期使用。

5、乙方对甲方用于广告等宣传等的各种形式的宣传作品不享有著作权，并承诺不会对有关的肖像权、名誉权、隐私权、著作权等问题，向甲方或甲方授权制作、使用、宣传的第三方提出任何权利要求。

6、本协议的甲方包括甲方及与甲方相关联的公司或其他组织、个人。

二、甲方的权利和义务

1、甲方有权要求乙方及时配合拍摄乙方的肖像供甲方使用。

2、按照本协议约定及时兑现给予乙方的优惠条件。

三、乙方的权利和义务

1、乙方有权按照本协议的约定，接受甲方因使用乙方的肖像而给予的各项优惠条件。

2、自本协议签订之日起，乙方按照甲方的要求的时间拍摄照片、影音视频、病患部位照片等肖像并发送给甲方，供甲方按本协议的约定使用。

3、甲方在需要乙方本人出面配合甲方进行商业或非商业宣传、交流等时，乙方应当给予全面配合。

四、优惠条件

1、乙方为甲方的患者，甲方应收取乙方的治疗费用　　元，优惠乙方　　元，甲方将优惠的　　元返还给乙方。

2、甲方返还给乙方的　　元，在本协议签订时，返还给乙方　　元，

乙方每次均按照本协议的约定配合甲方拍摄肖像并发送给甲方供甲方使用后，满　　年，甲方将余下的　　元，返还给乙方。

3、自本协议签订之日起，乙方两次或以上，未按照甲方要求的时间和数量拍摄并发送照片、影音视频等肖像给甲方或者乙方以任何方式防碍甲方使用其肖像或拒绝出面配合甲方的宣传活动等，甲方有权取消给予乙方的全部优惠，乙方应将甲方已返还的　　元交还给甲方，余下的　　元，甲方不再返还给乙方。

五、争议解决的方式

因履行本协议产生的所有争议，双方通过协商解决，协商不成，双方均可向甲方所在地的人民法院提起诉讼。

六、本协议一式两份，双方各执一份，签字、盖章后生效。

甲方：　　　　　　　　　　　　乙方：郁文鹏

代表人：刘素玉

2022年3月15日
