# Supplementary material for: Clinical validation and study of stem cell transplantation in treatment of vitiligo
Source: Arch Dermatol Res. 2023 Sep 7;315(10):2983–4. doi: 10.1007/s00403-023-02692-5 (PMC10615963; doi:10.1007/s00403-023-02692-5)
Supplement: Supplementary file 2 — Supplementary file2 (PDF 51673 KB) [file 403_2023_2692_MOESM2_ESM.pdf]

Cases of stem cell therapy for vitiligo

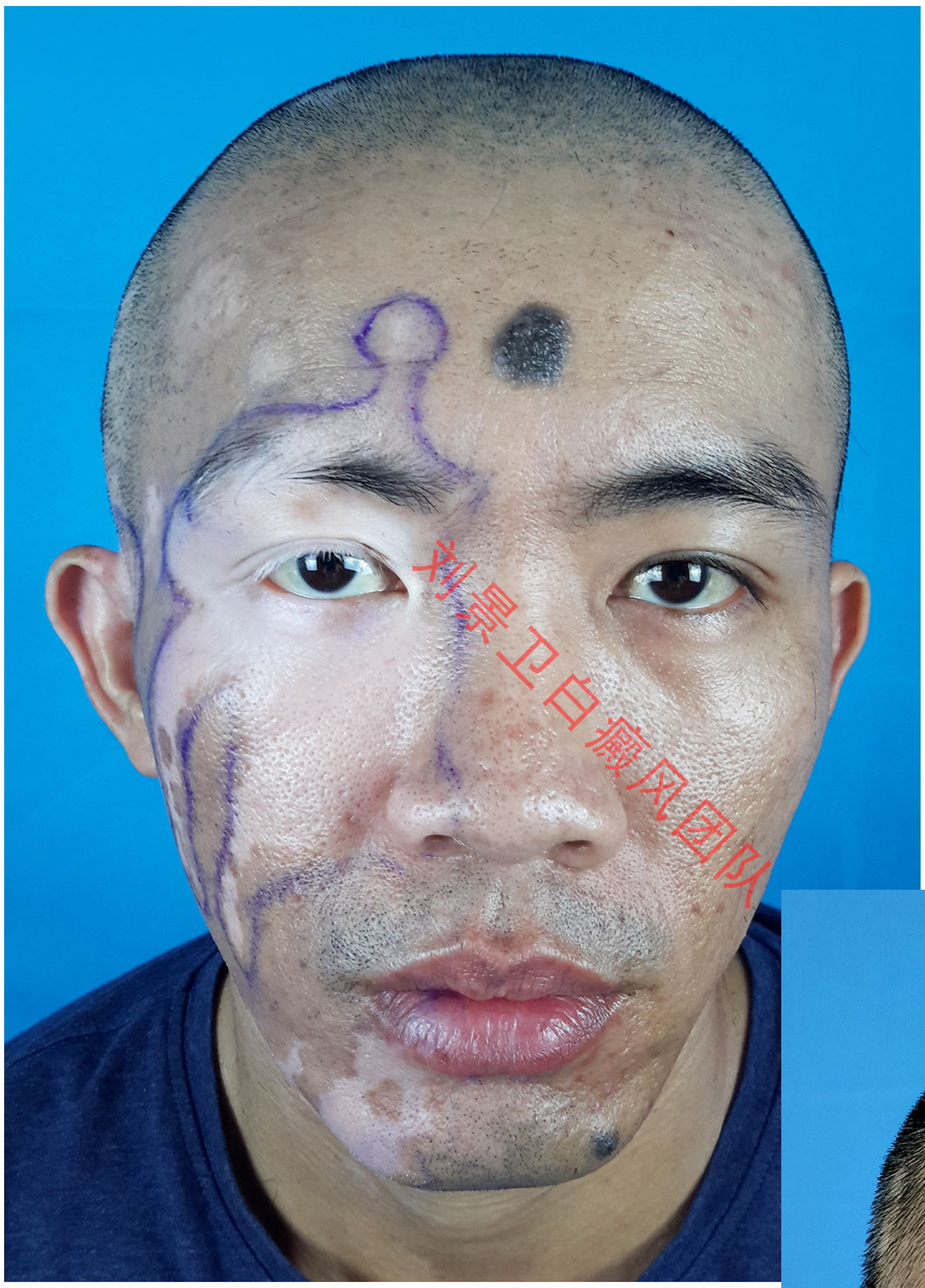

Before treatment

After treatment

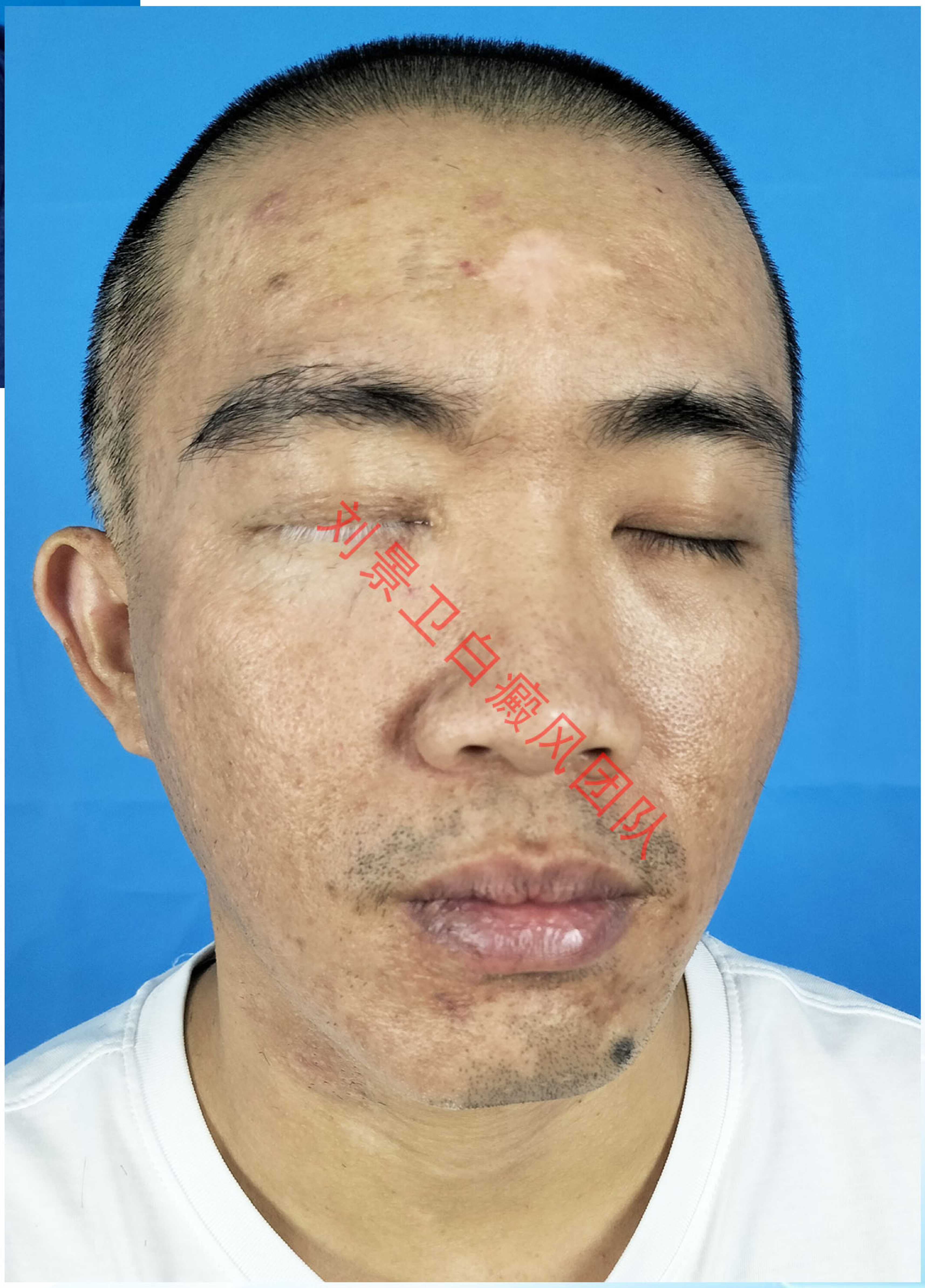

Cases of stem cell therapy for vitiligo

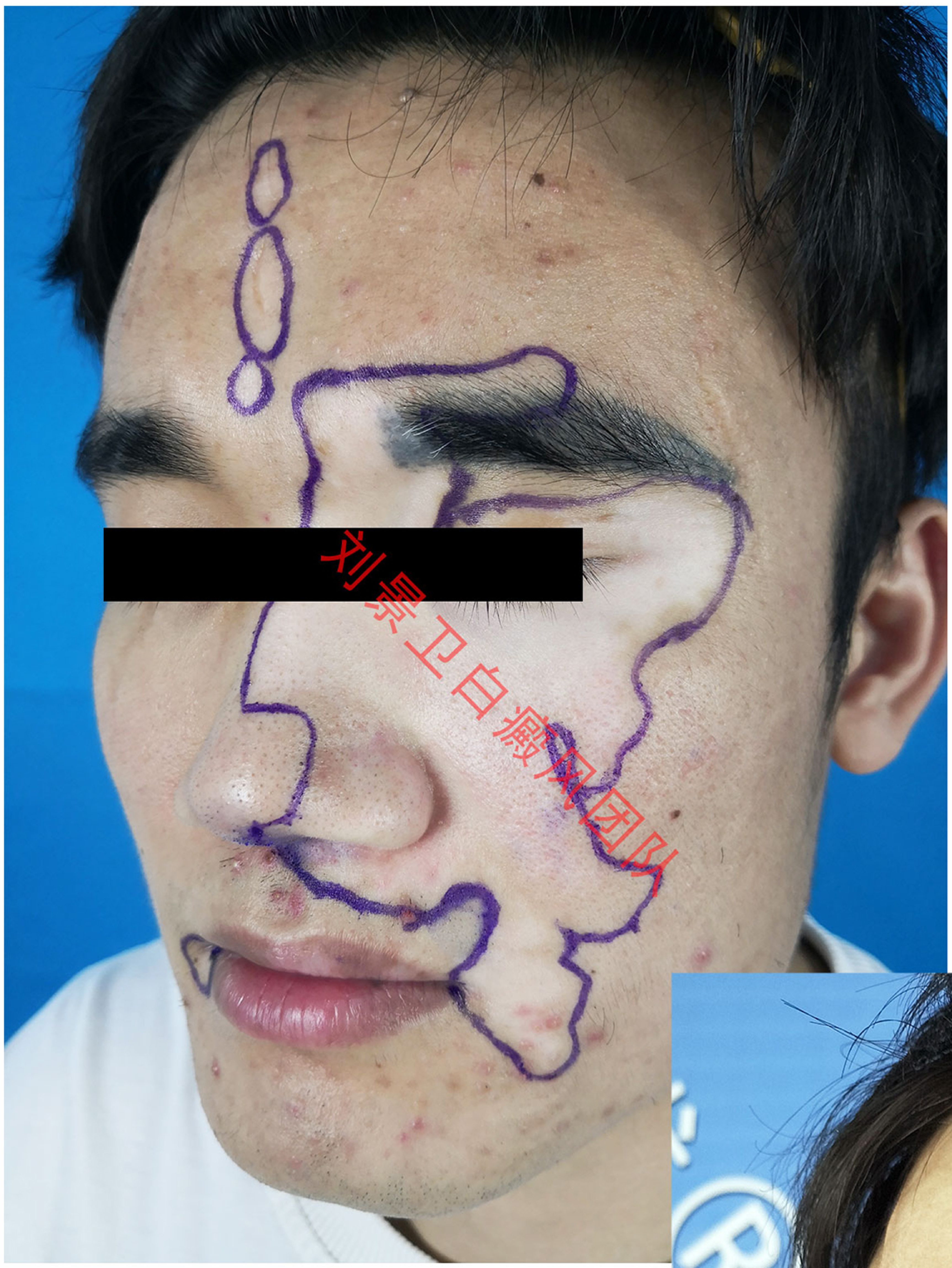

Before treatment

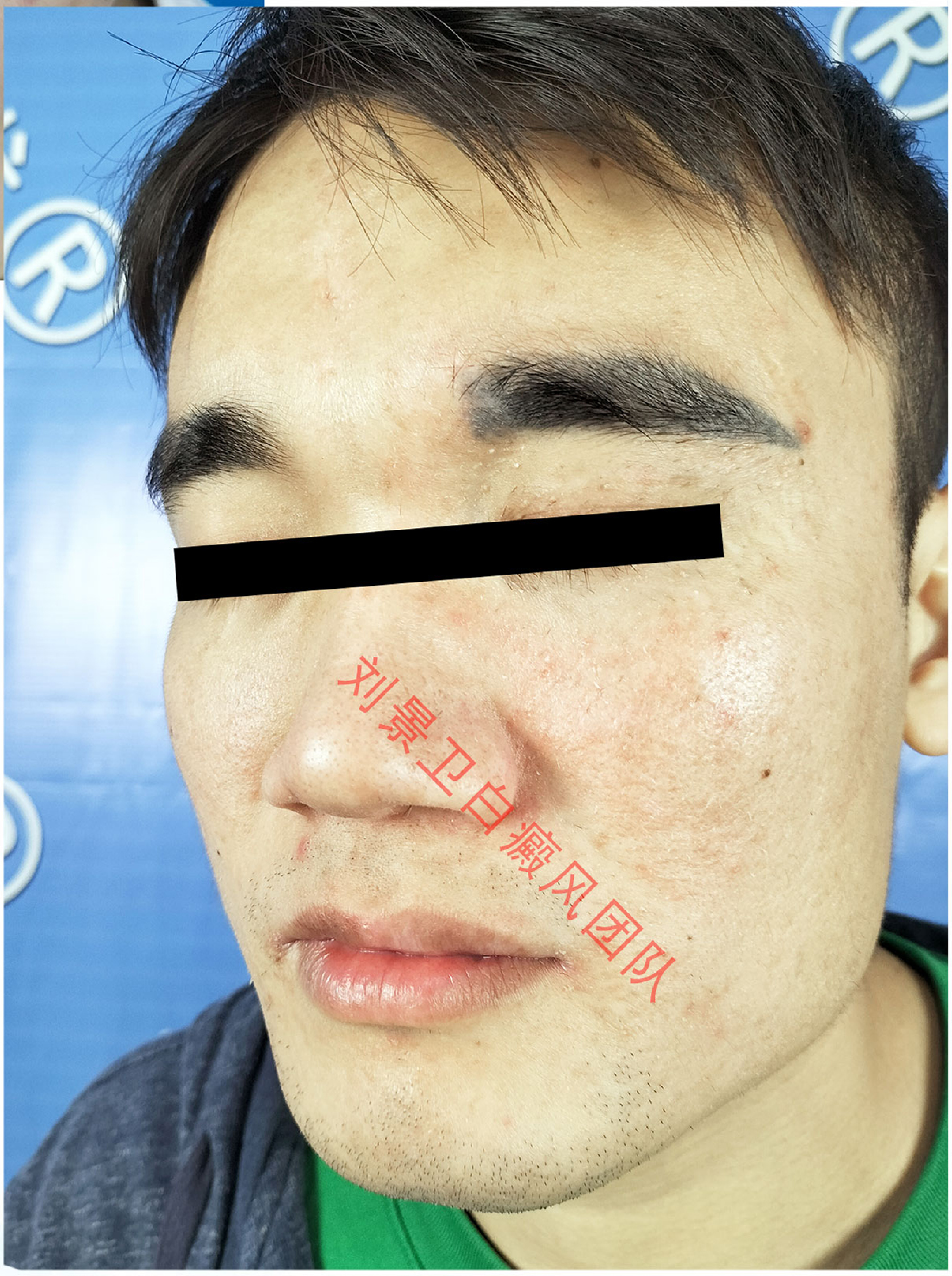

After treatment

Cases of stem cell therapy for vitiligo

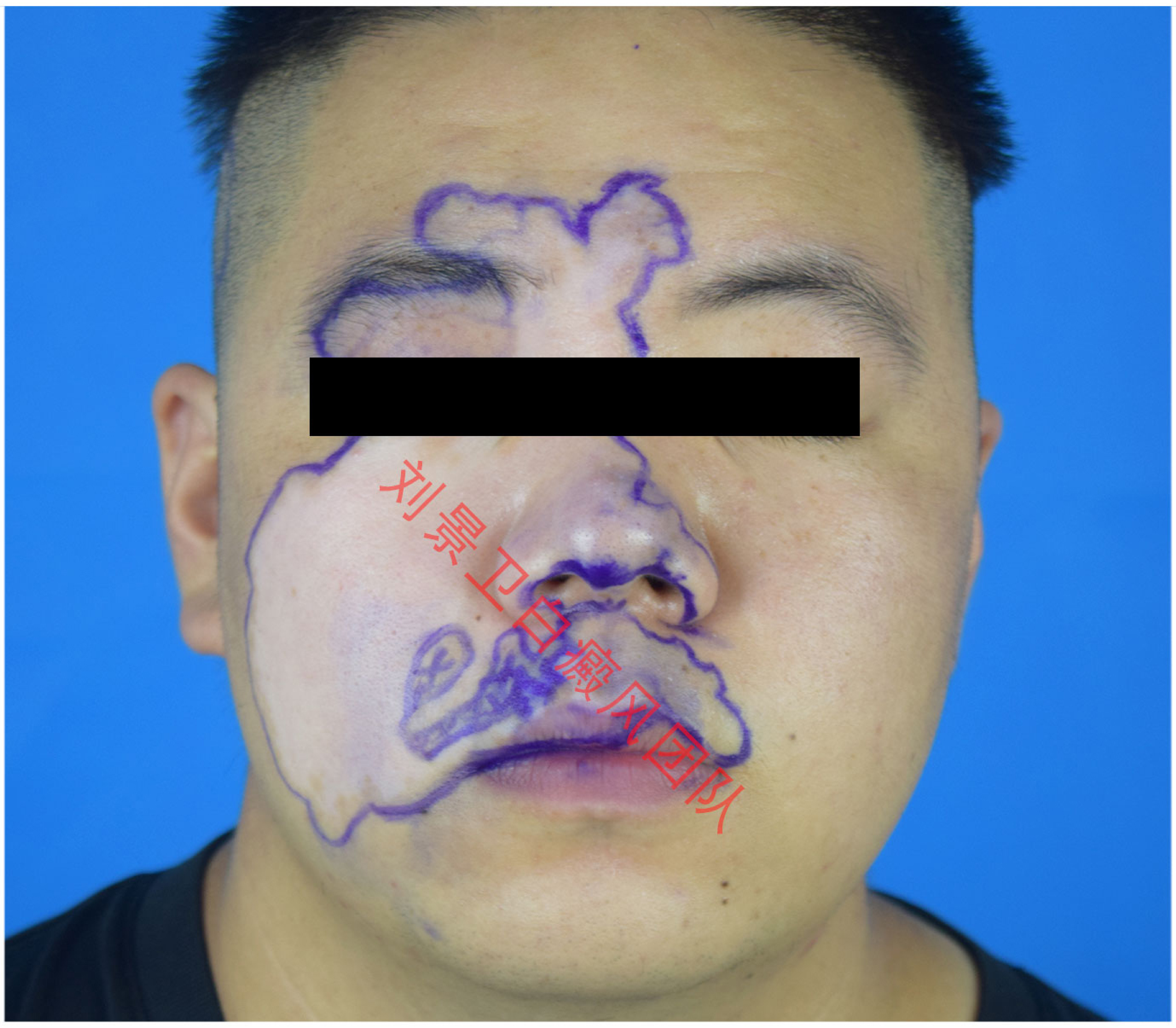

Before treatment

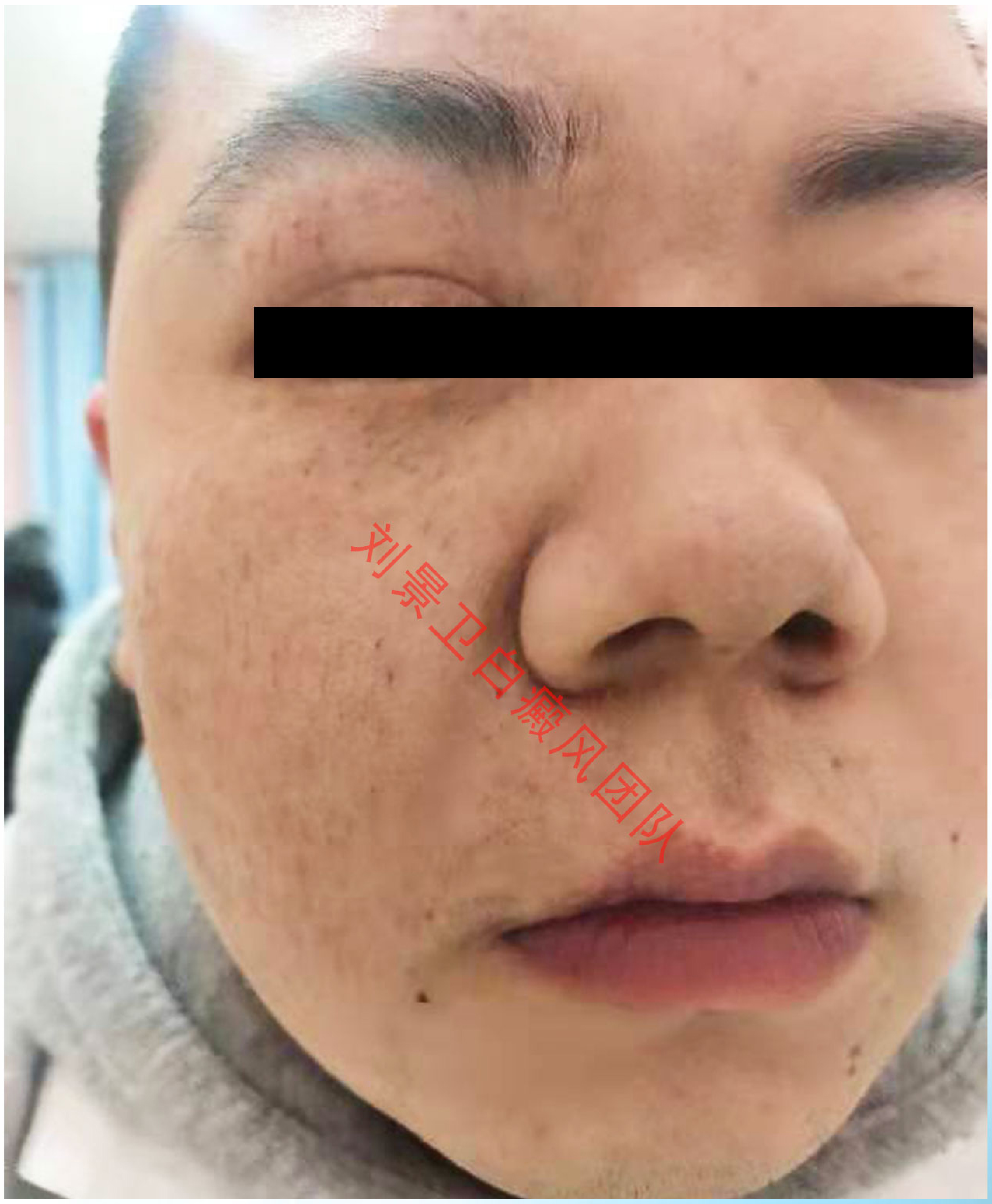

After treatment

Cases of stem cell therapy for vitiligo

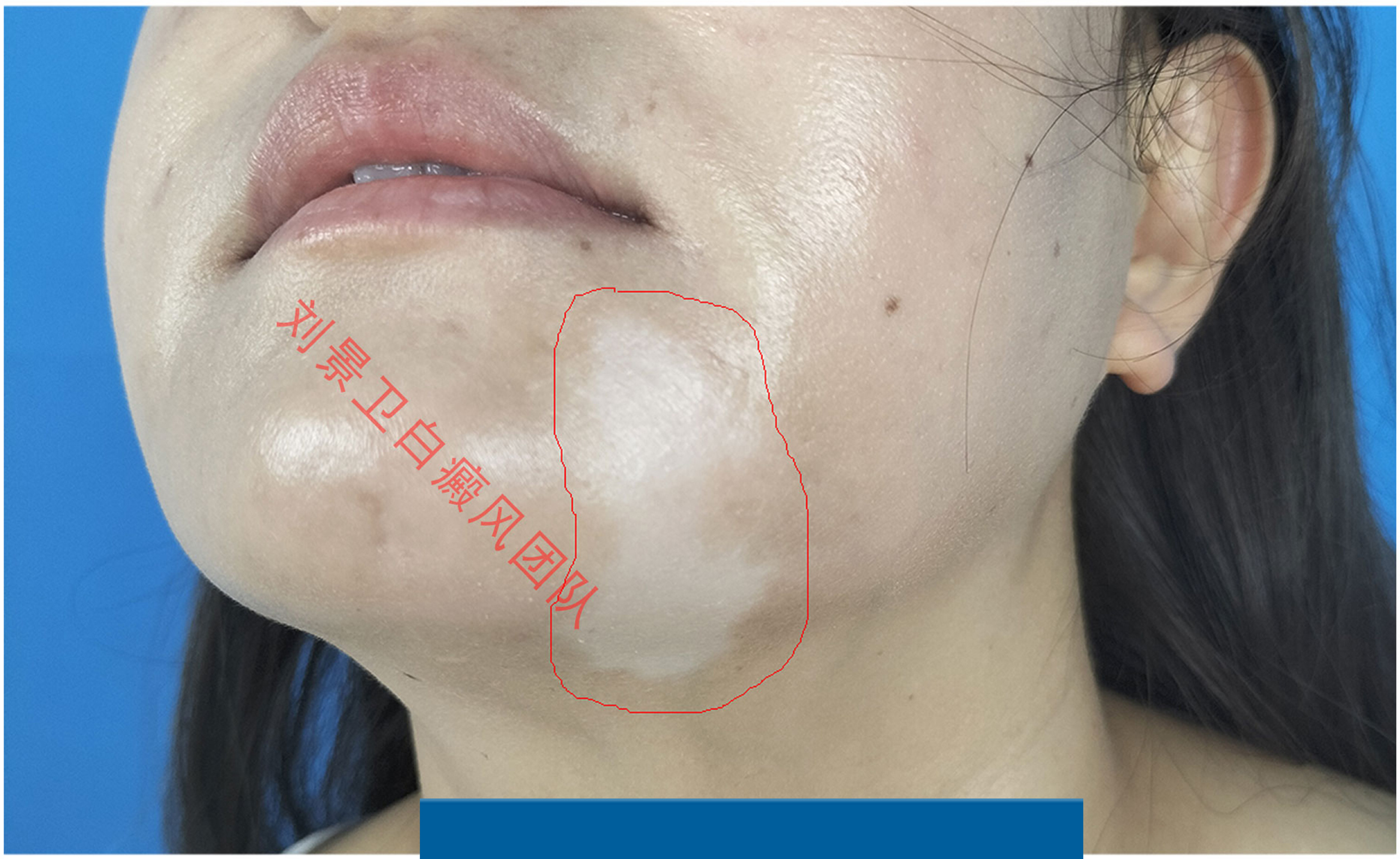

Before treatment

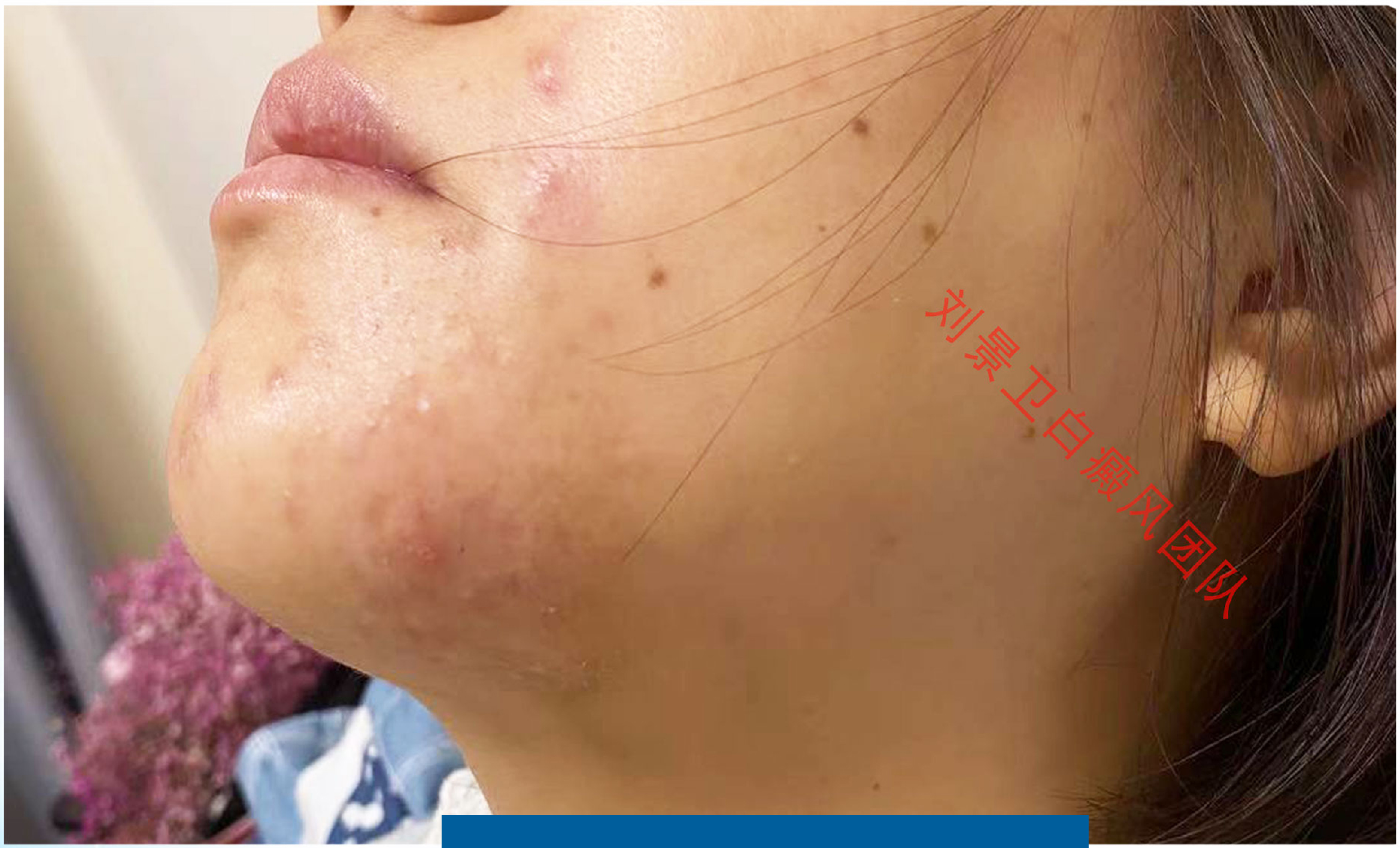

After treatment

Cases of stem cell therapy for vitiligo

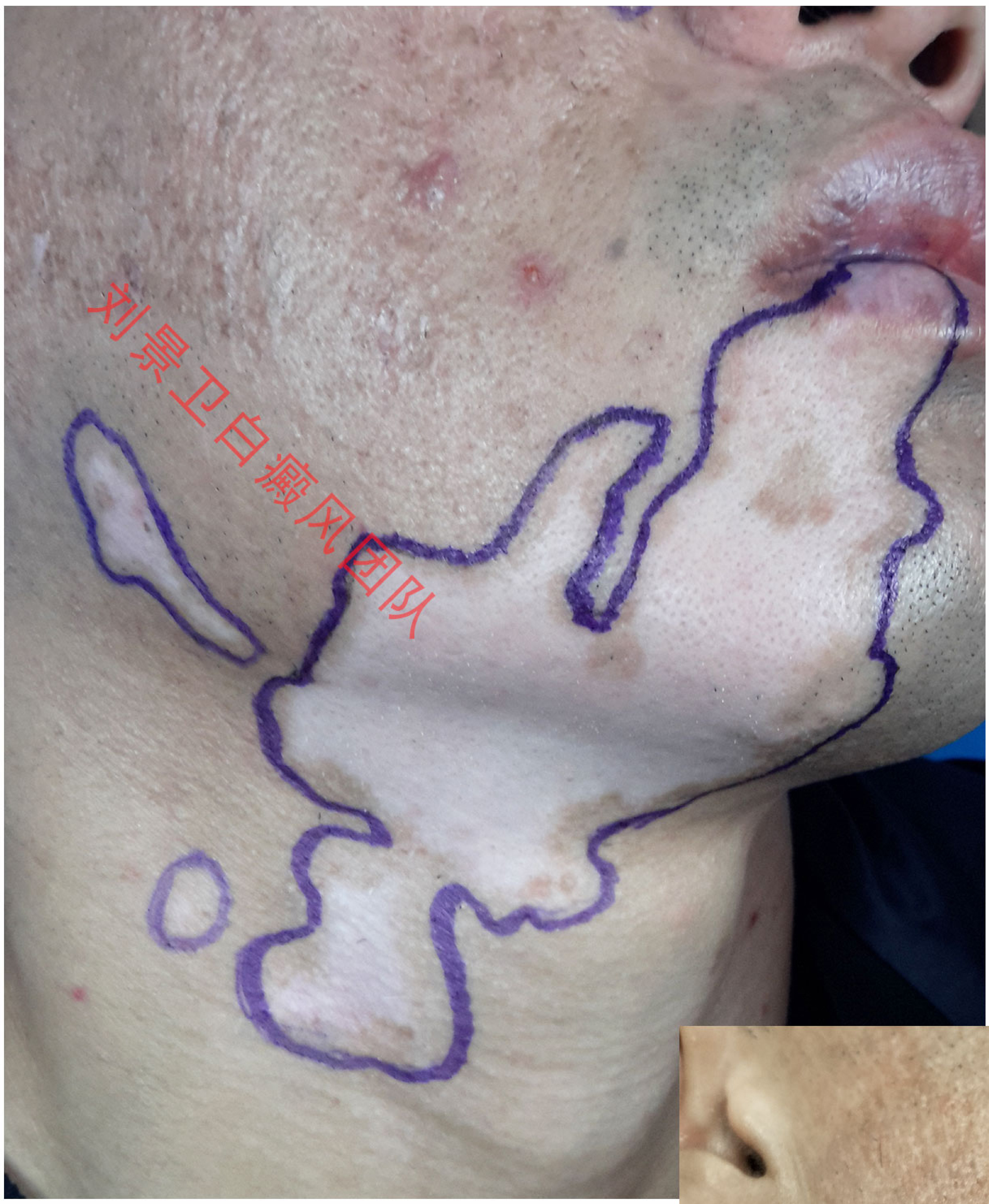

Before treatment

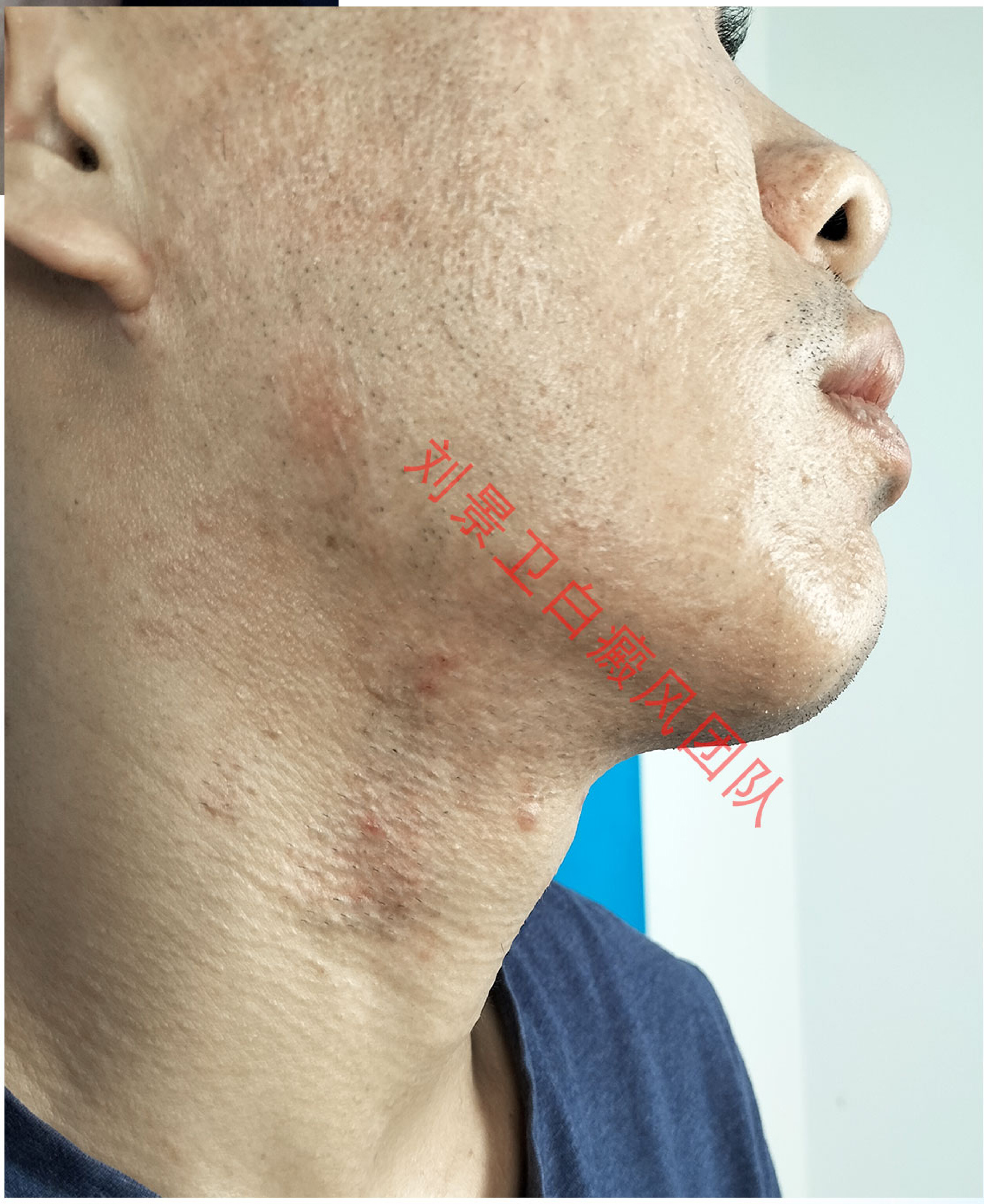

After treatment

Cases of stem cell therapy for vitiligo

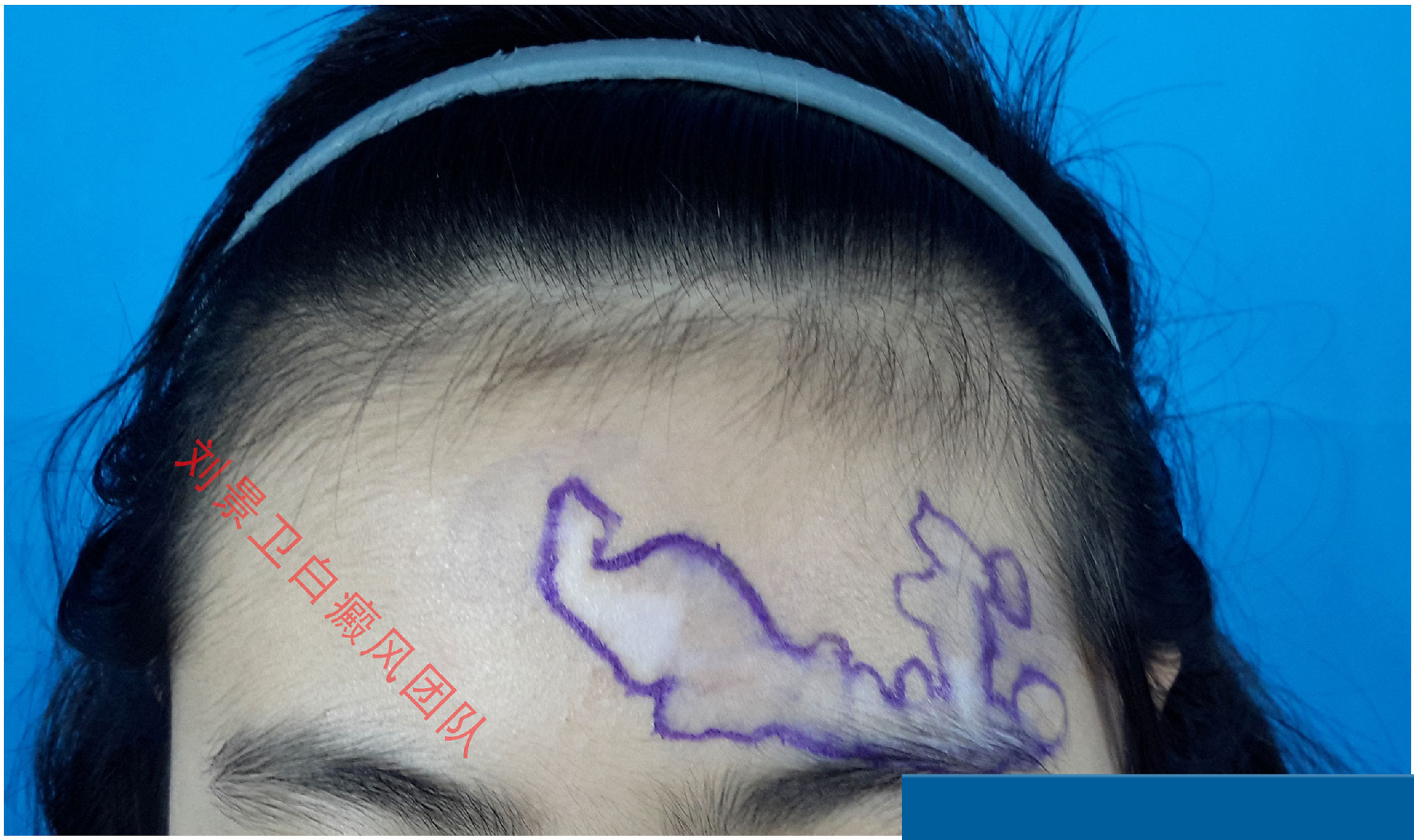

Before treatment

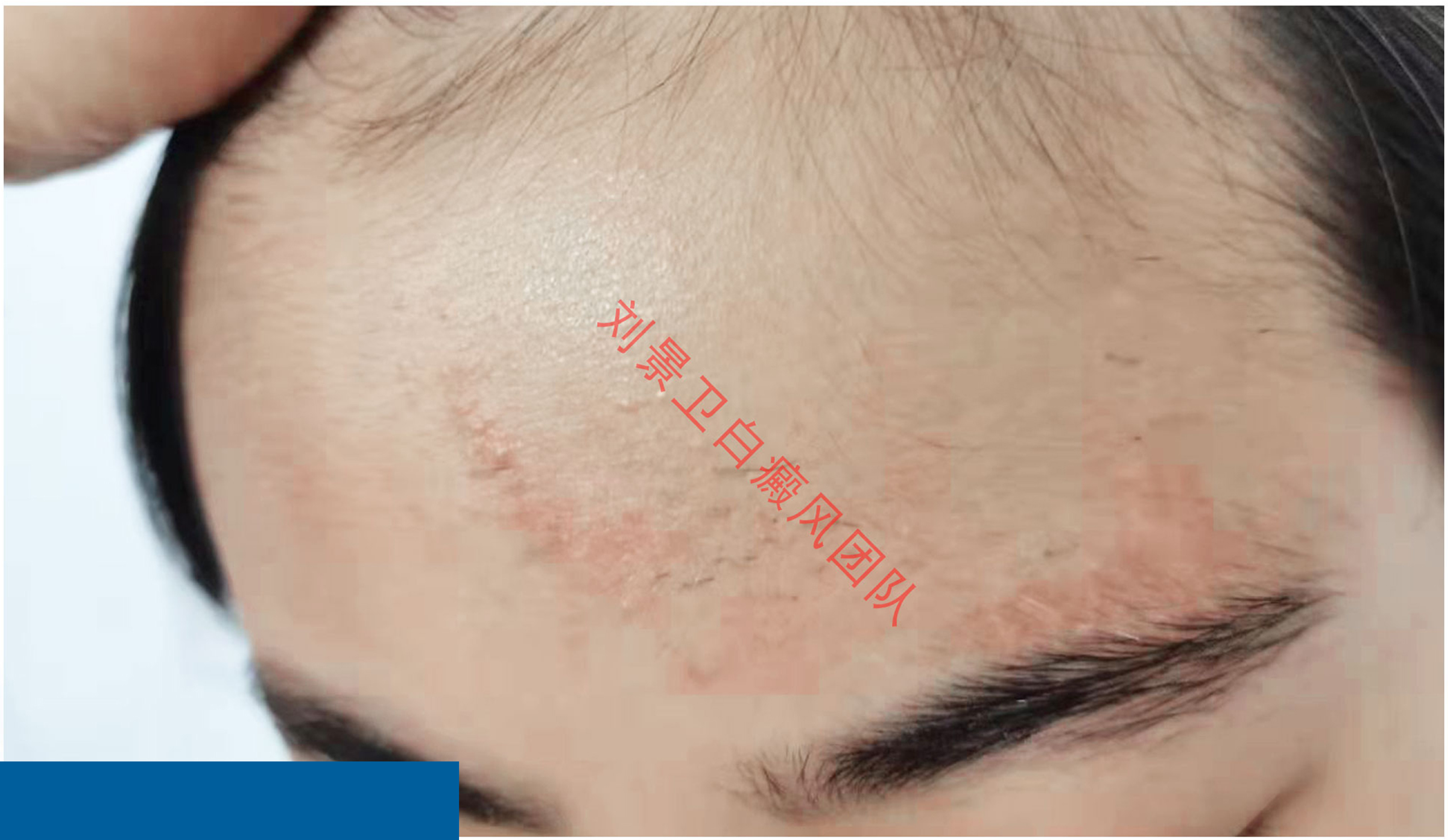

After treatment

Cases of stem cell therapy for vitiligo

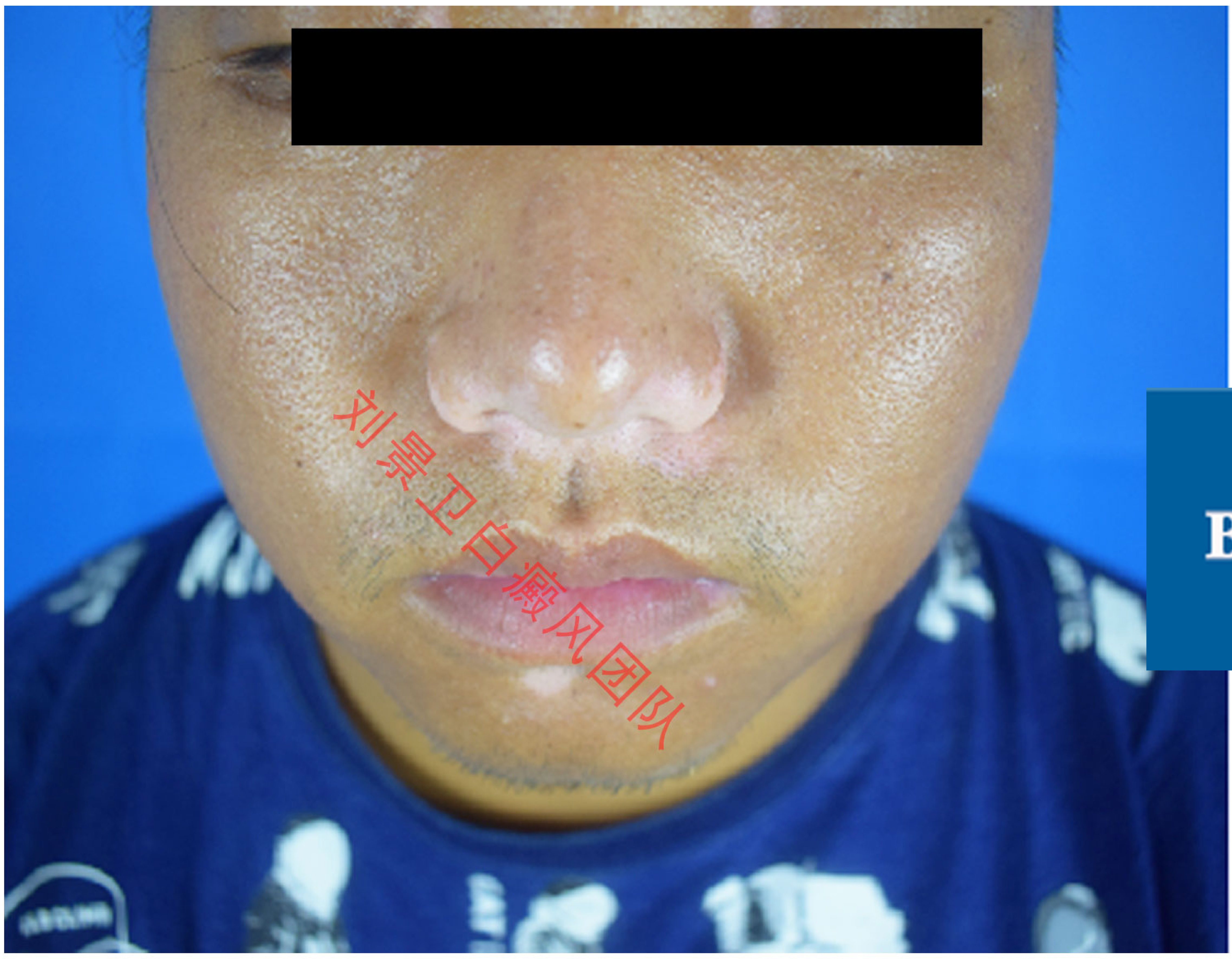

Before treatment

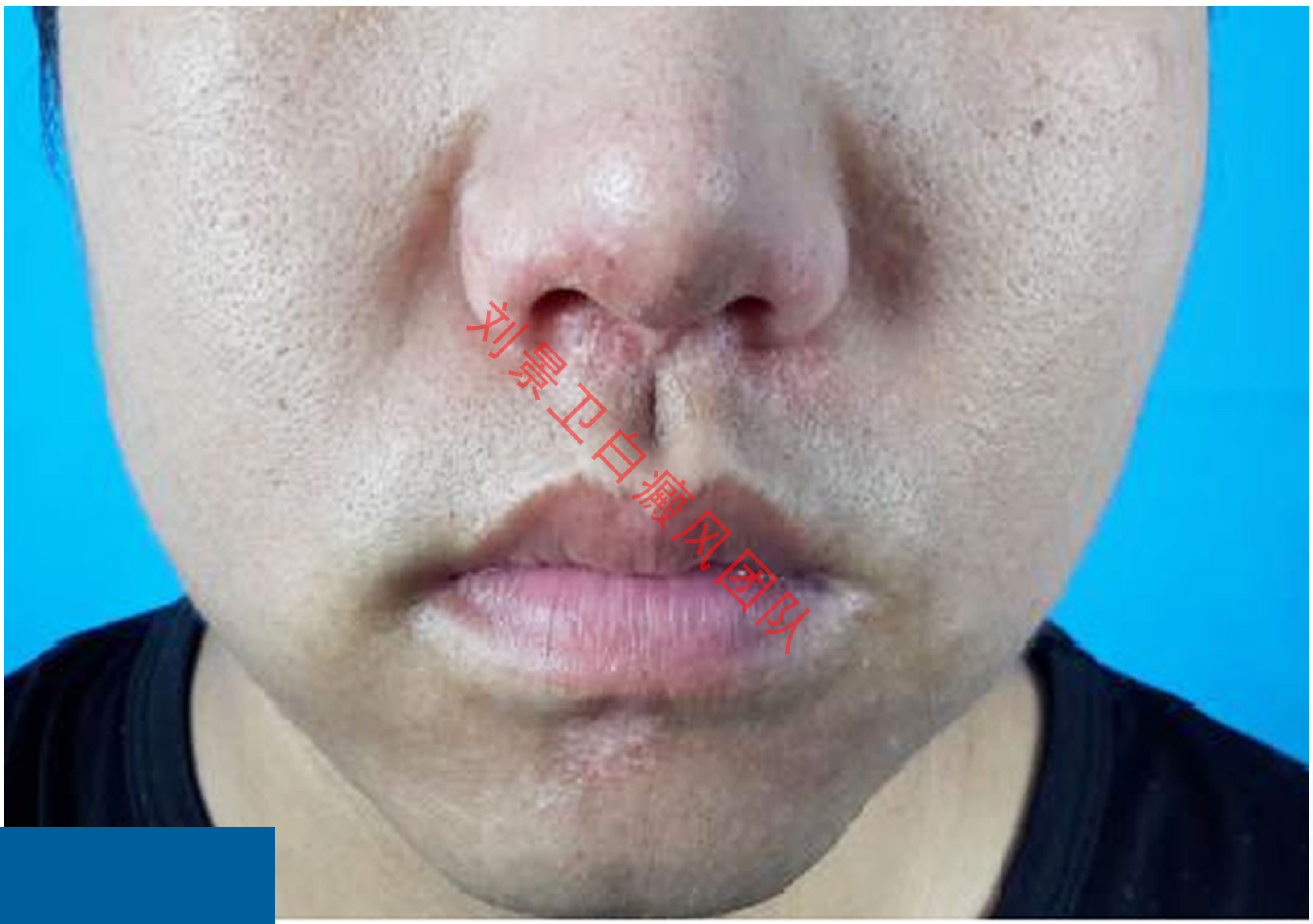

After treatment

Cases of stem cell therapy for vitiligo

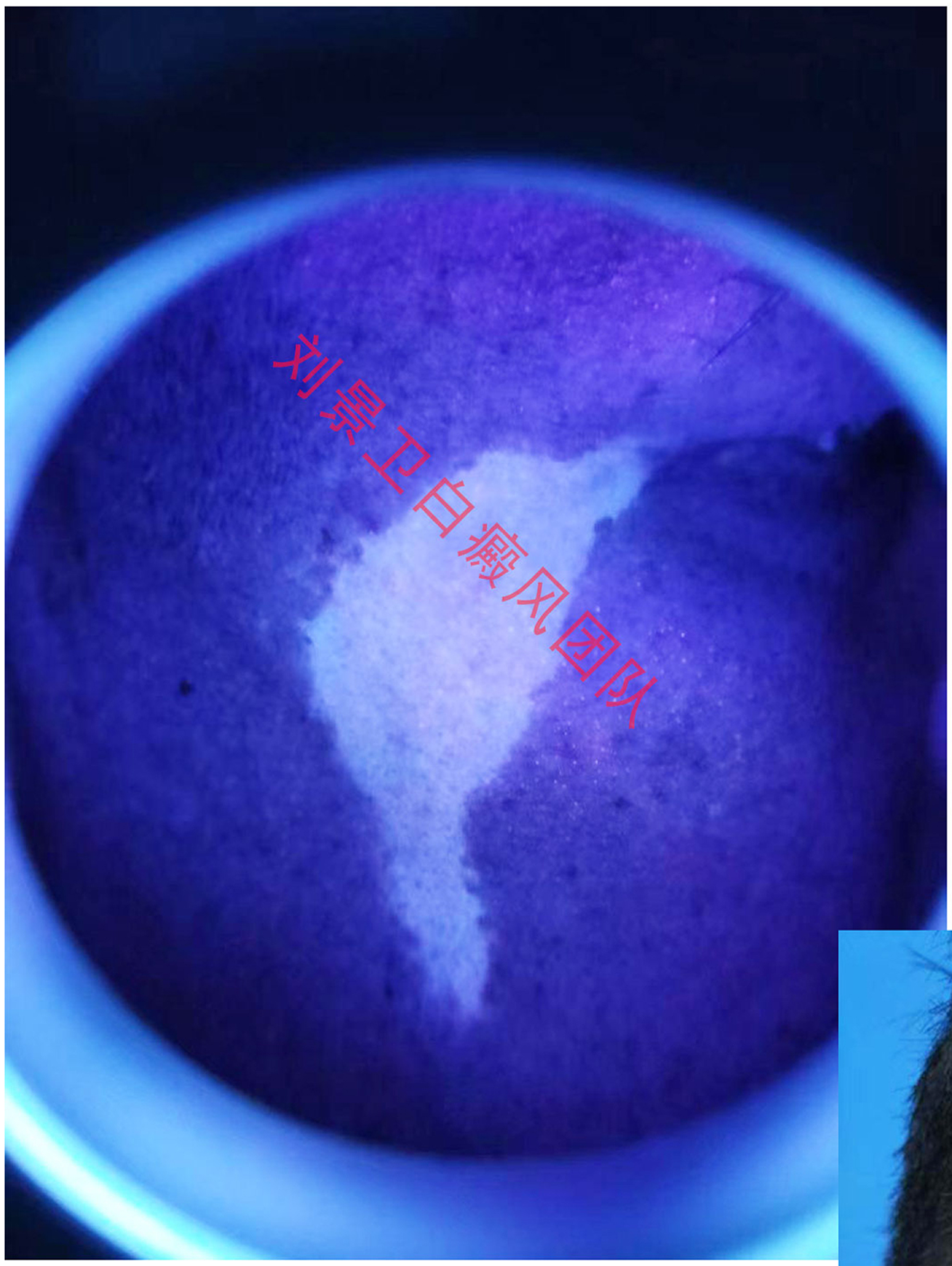

Before treatment

After treatment

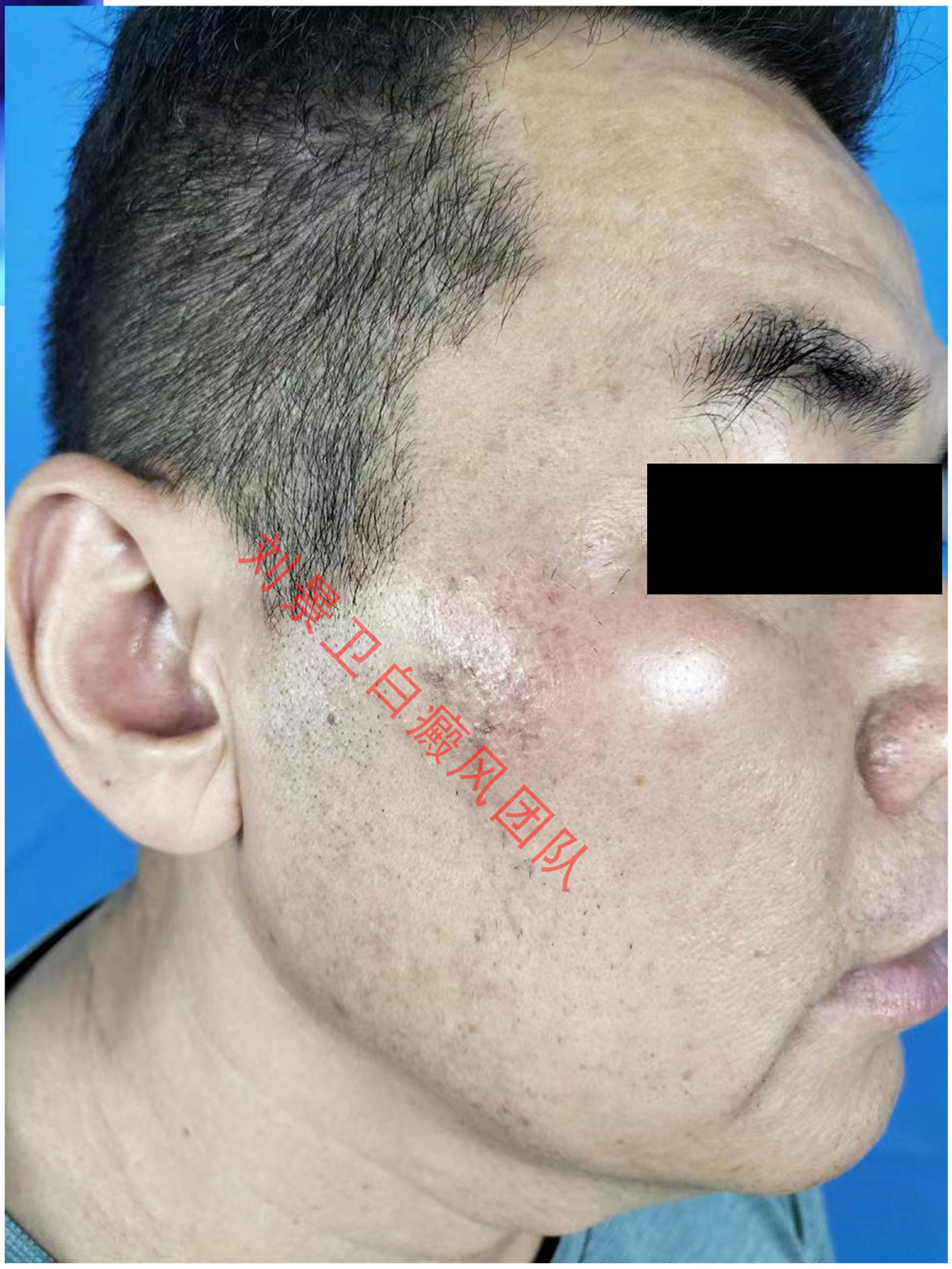

Cases of stem cell therapy for vitiligo

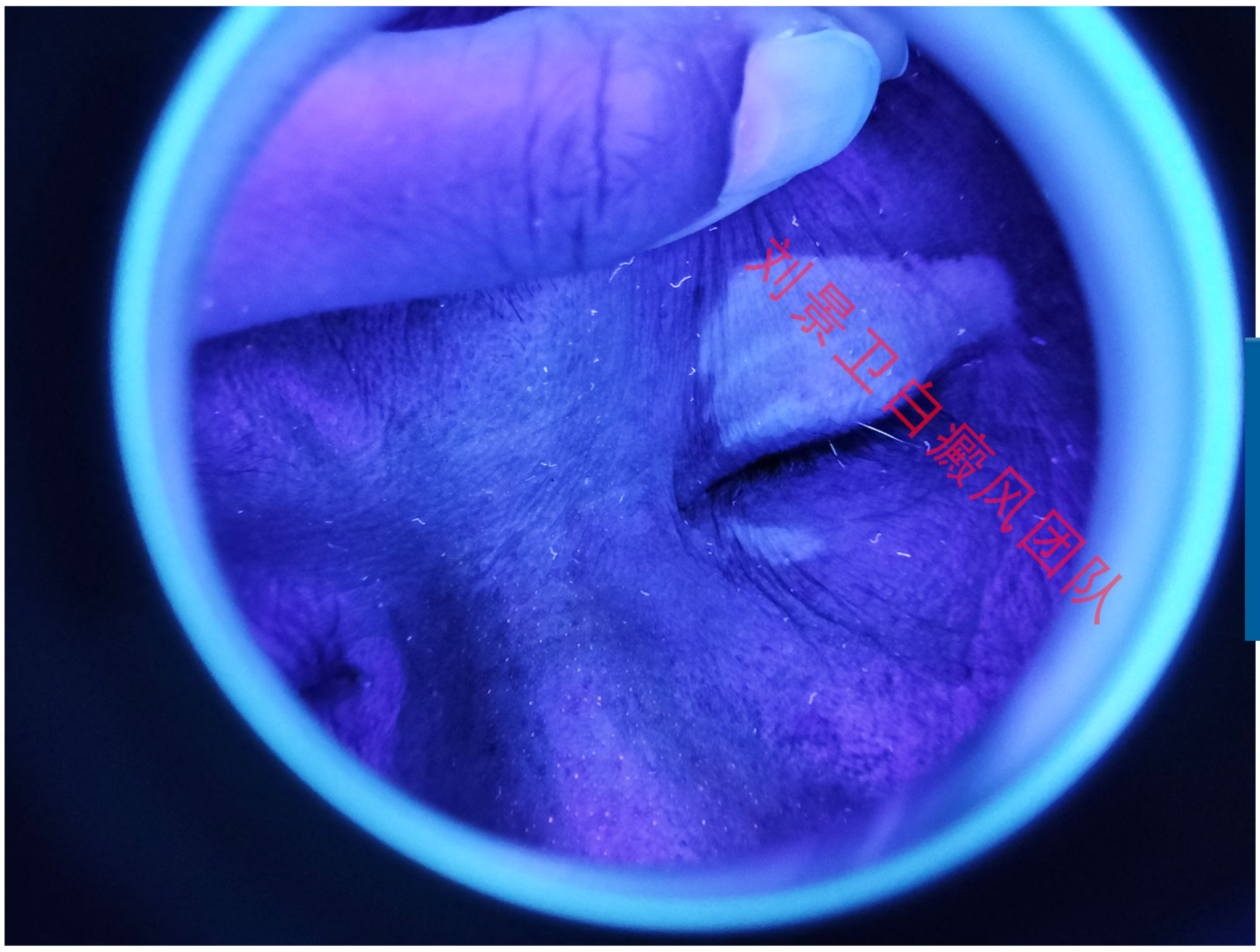
刘景卫白癜风团队

Before treatment

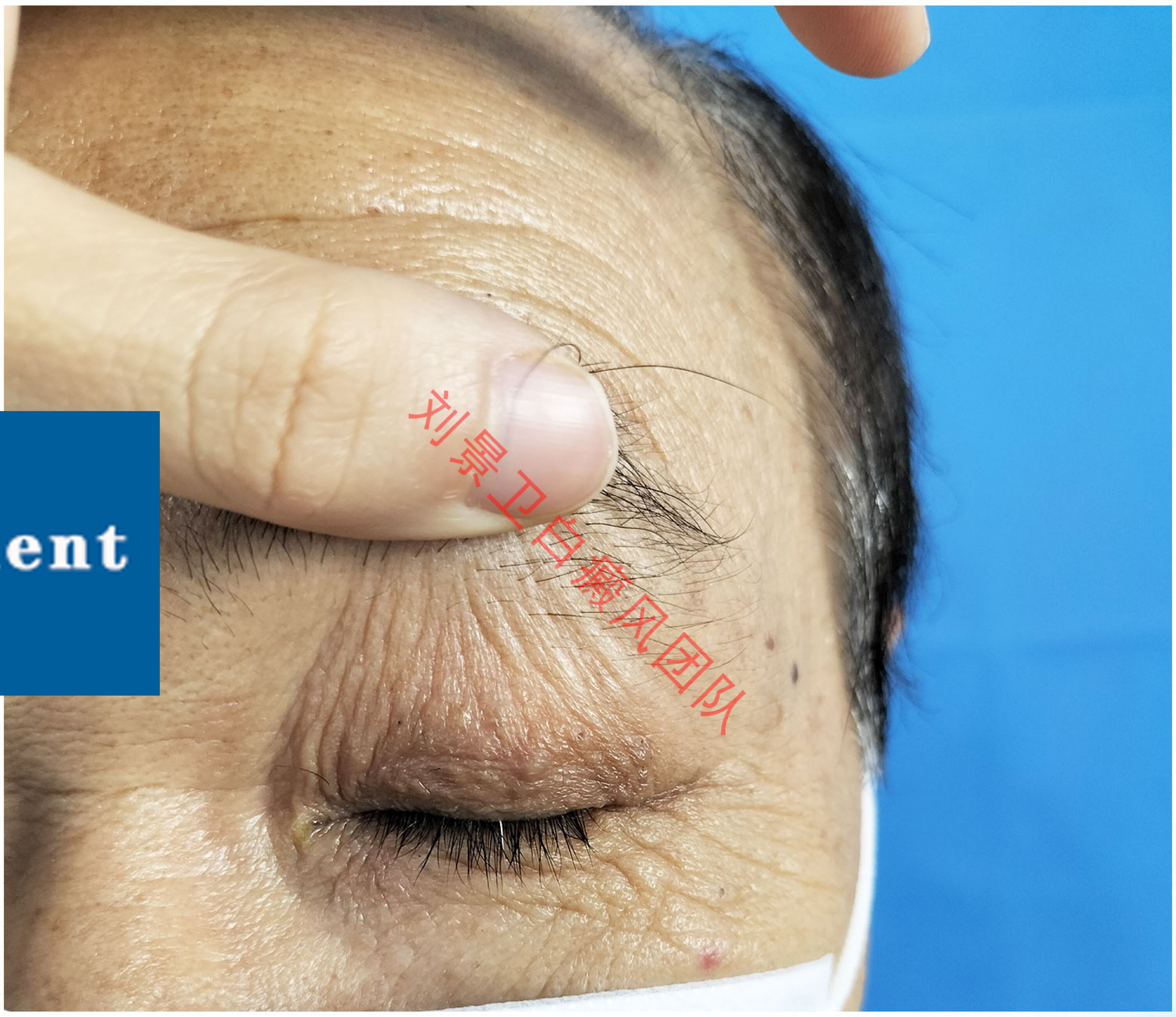
刘景卫白癜风团队

After treatment

Cases of stem cell therapy for vitiligo

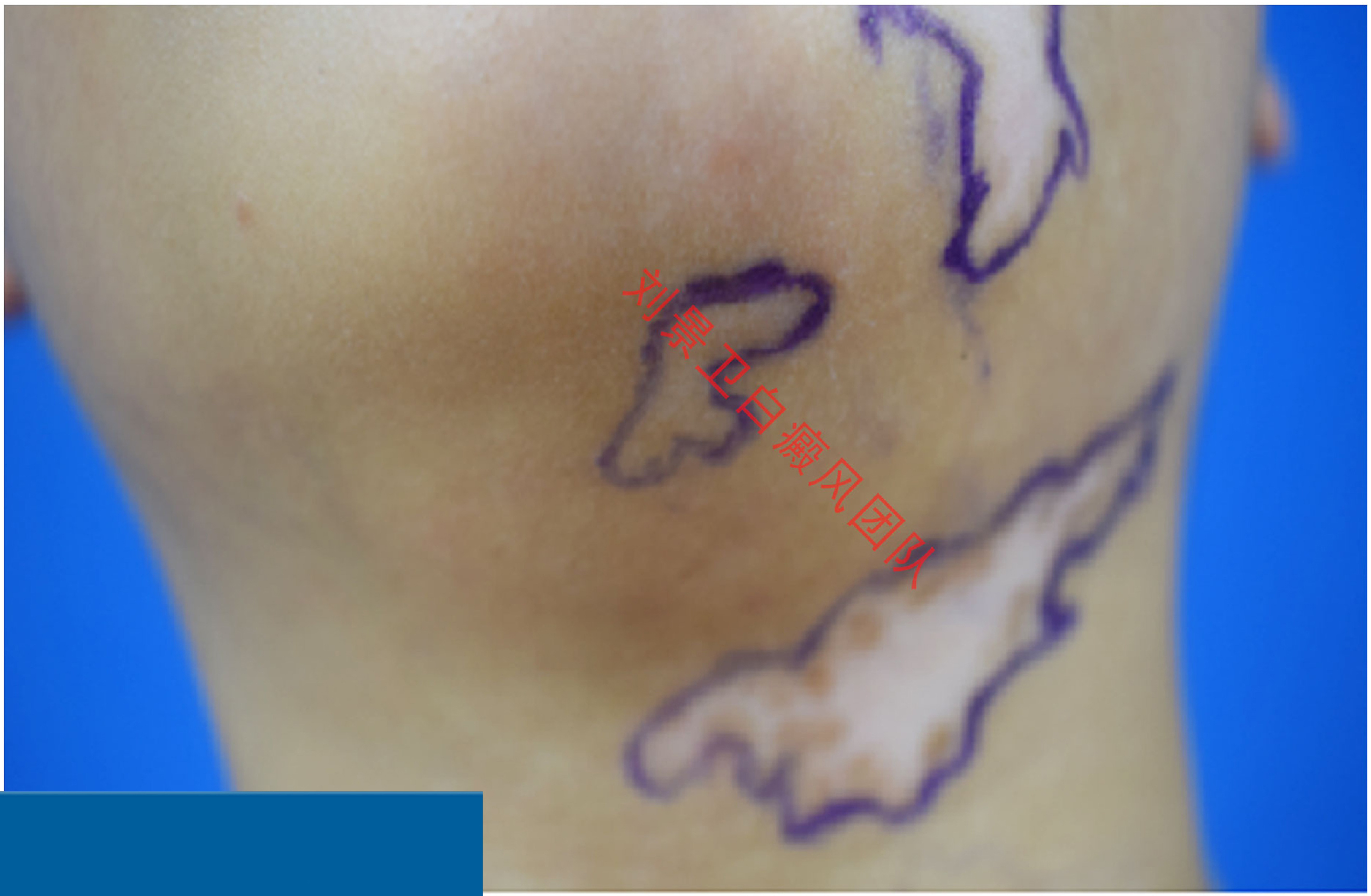
刘景卫白癜风团队

Before treatment

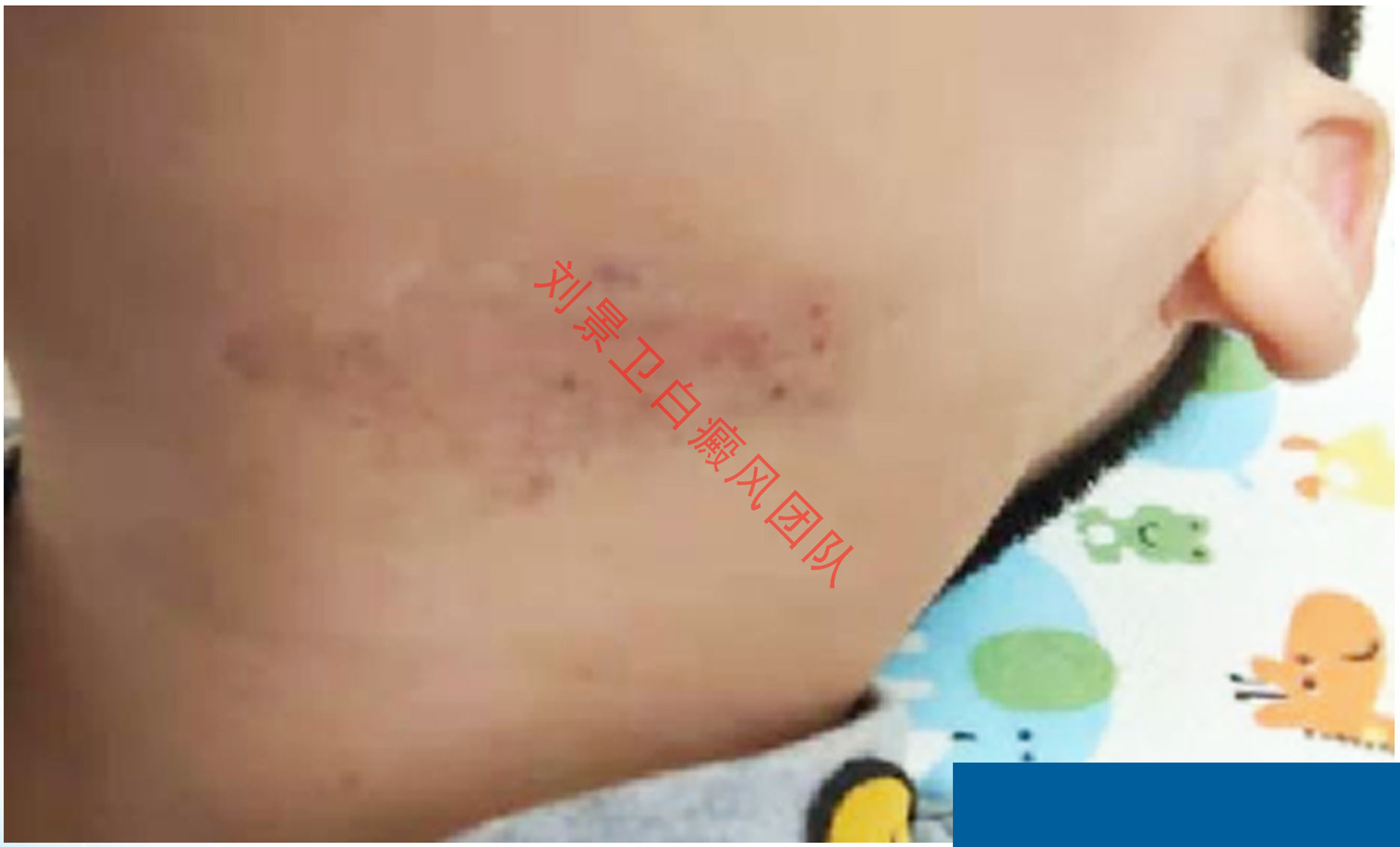
刘景卫白癜风团队

After treatment

Cases of stem cell therapy for vitiligo

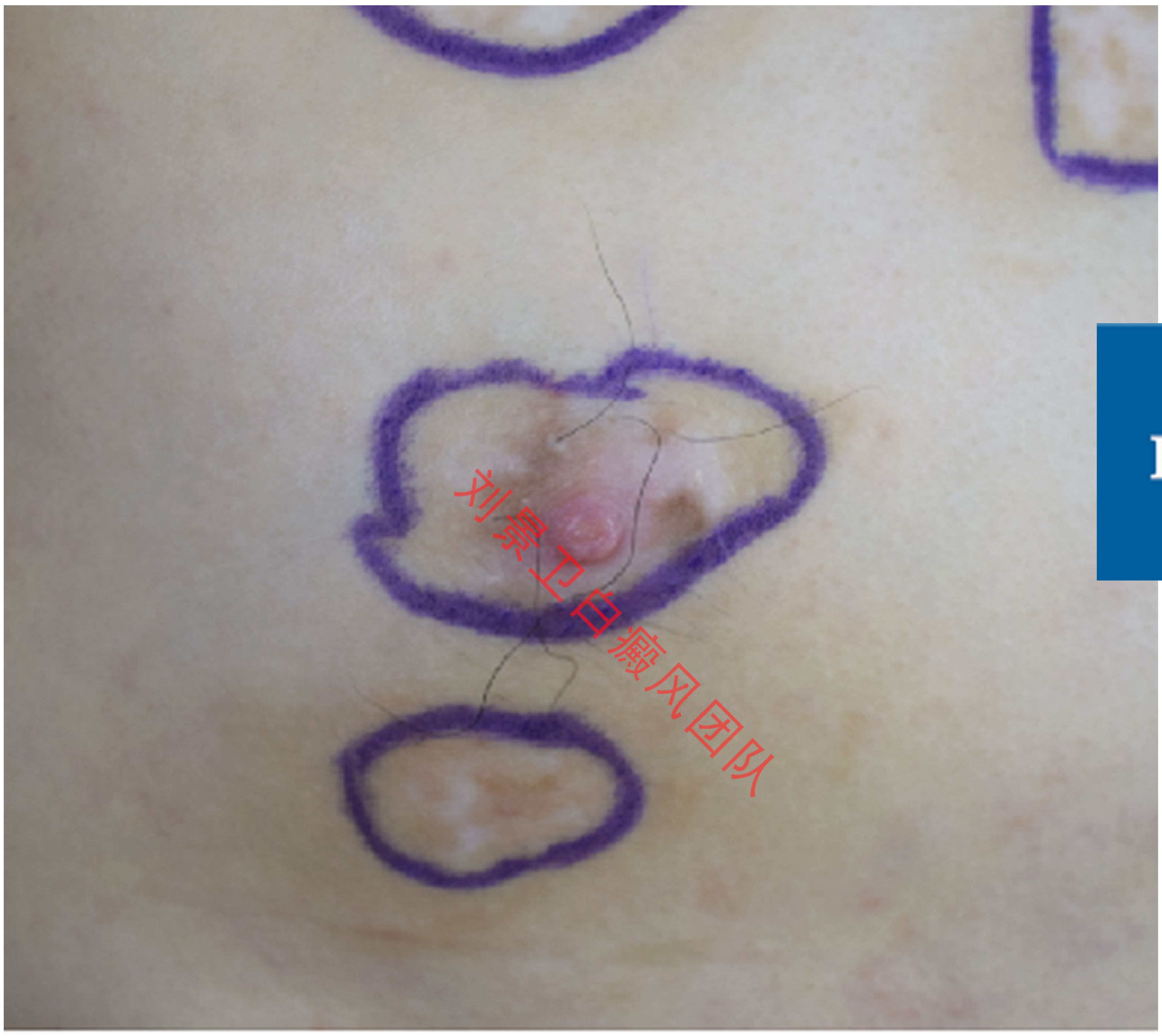

Before treatment

After treatment

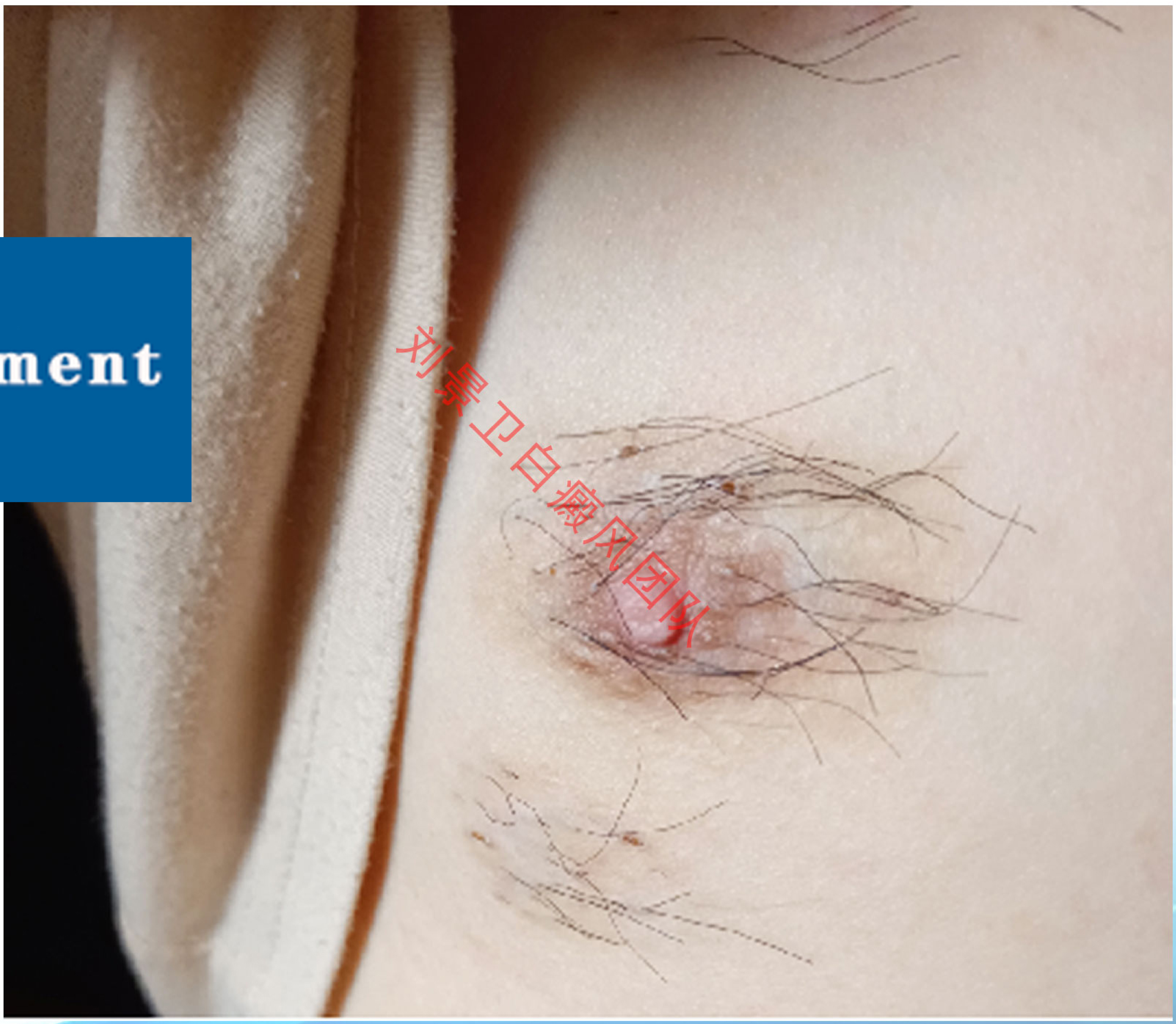

Cases of stem cell therapy for vitiligo

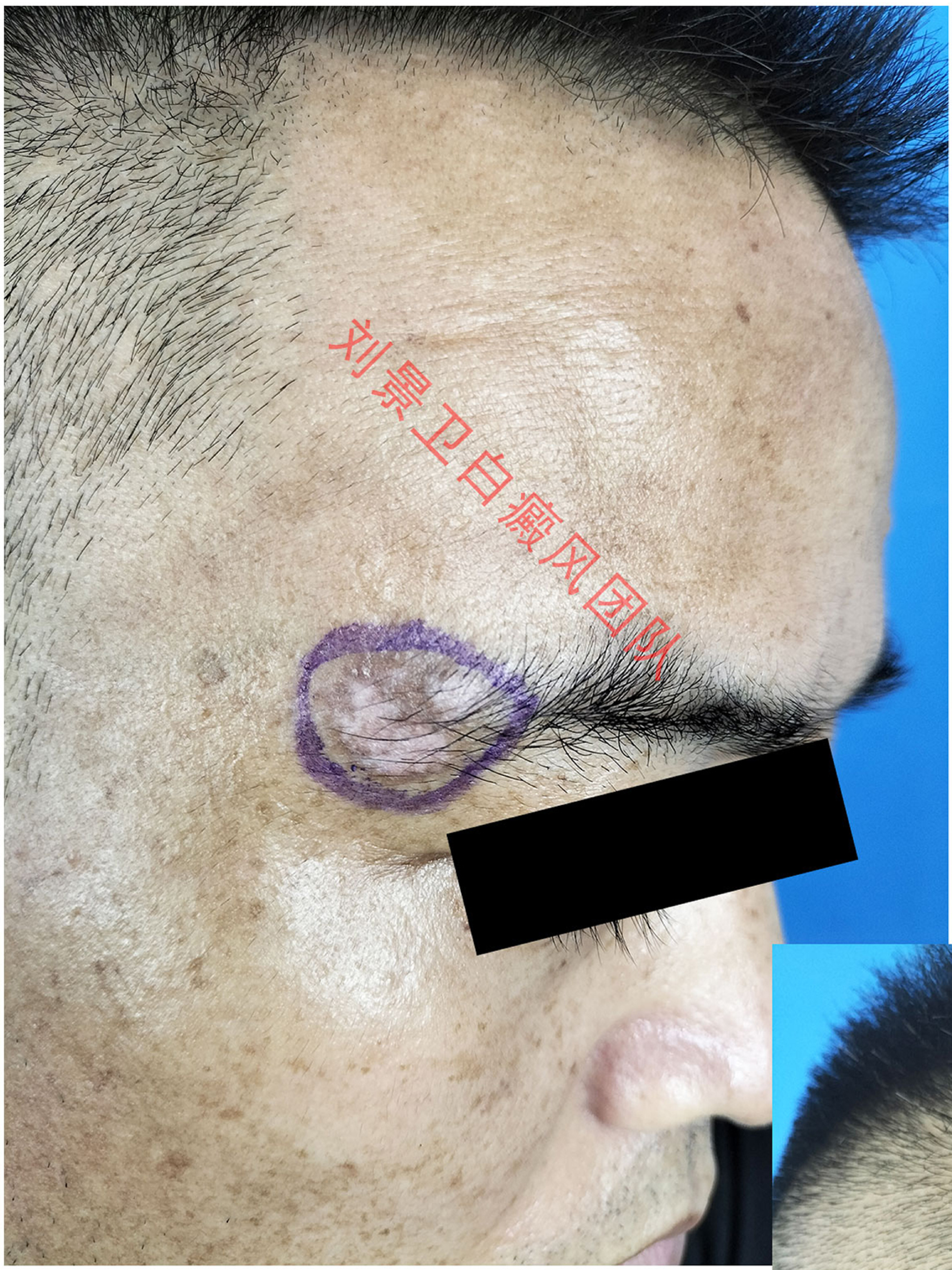

Before treatment

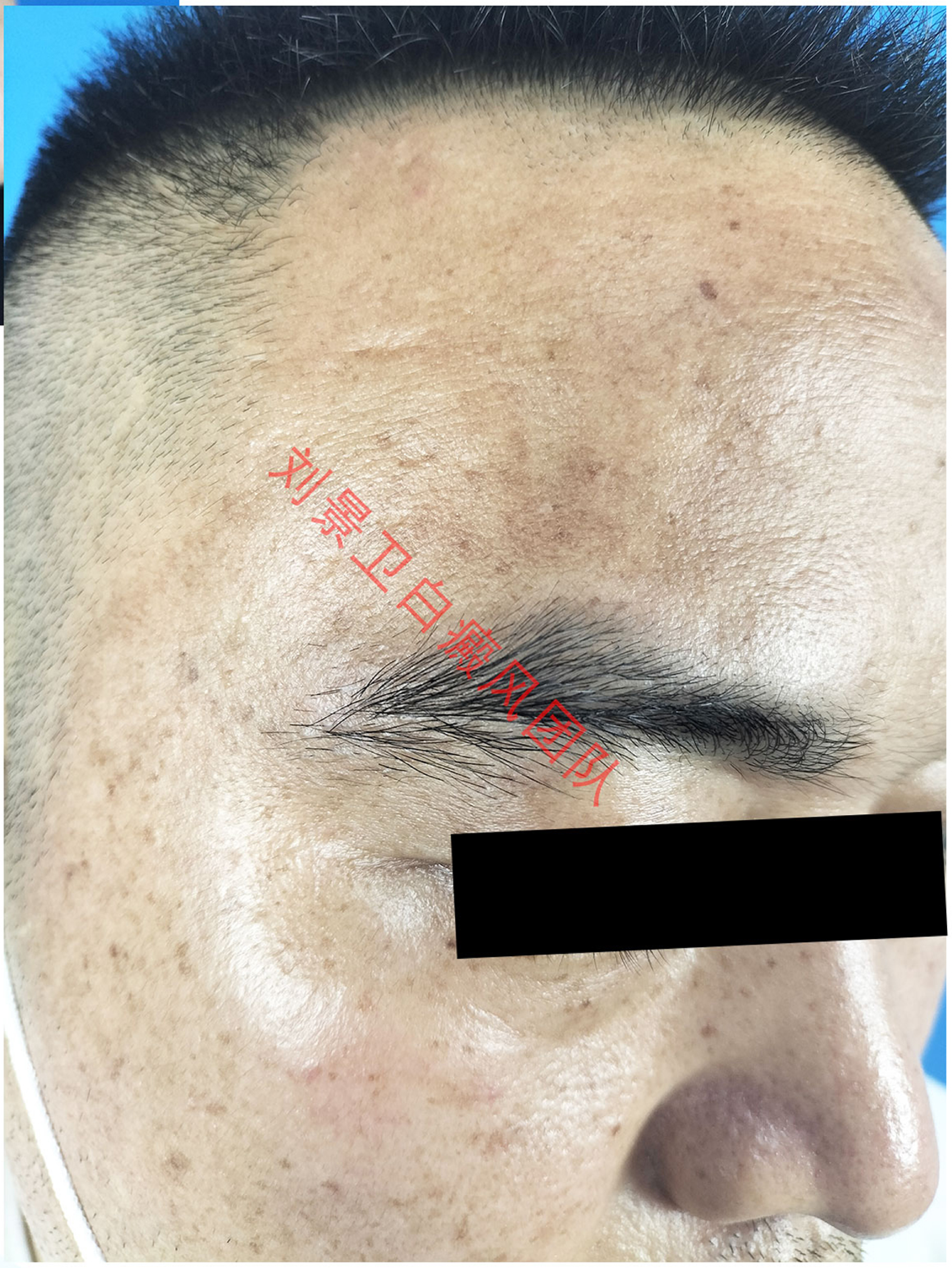

After treatment

Cases of stem cell therapy for vitiligo

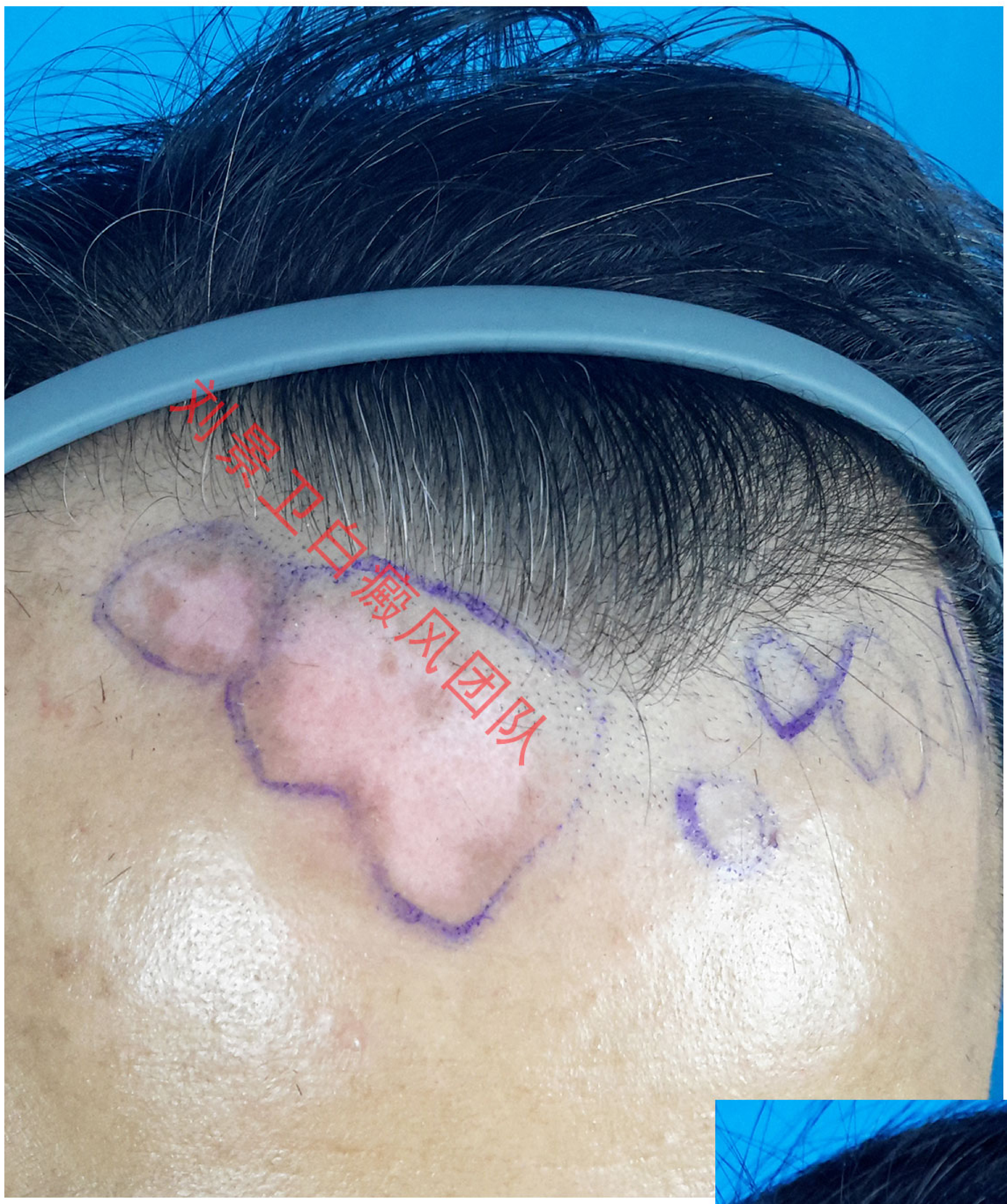

Before treatment

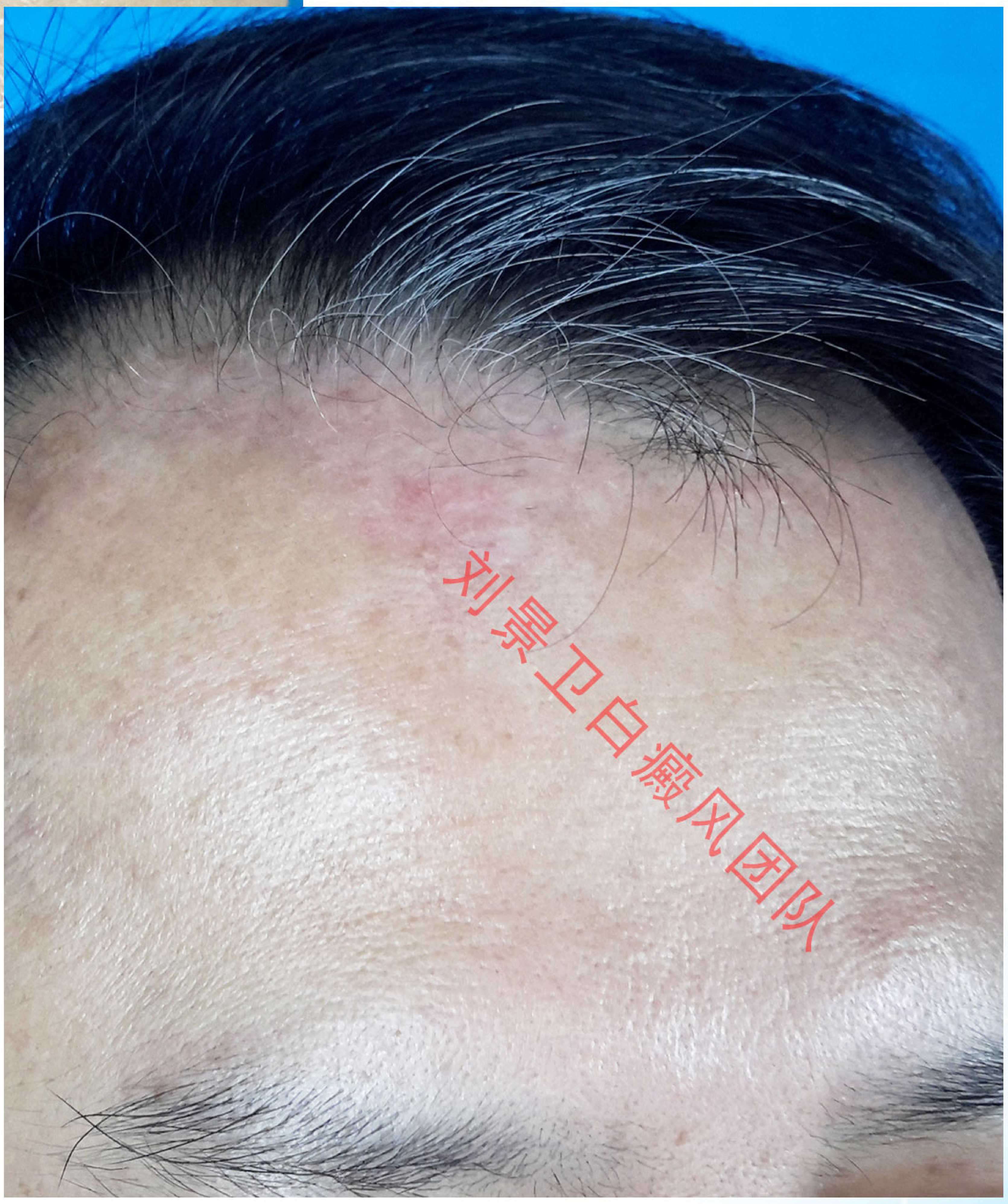

After treatment

Cases of stem cell therapy for vitiligo

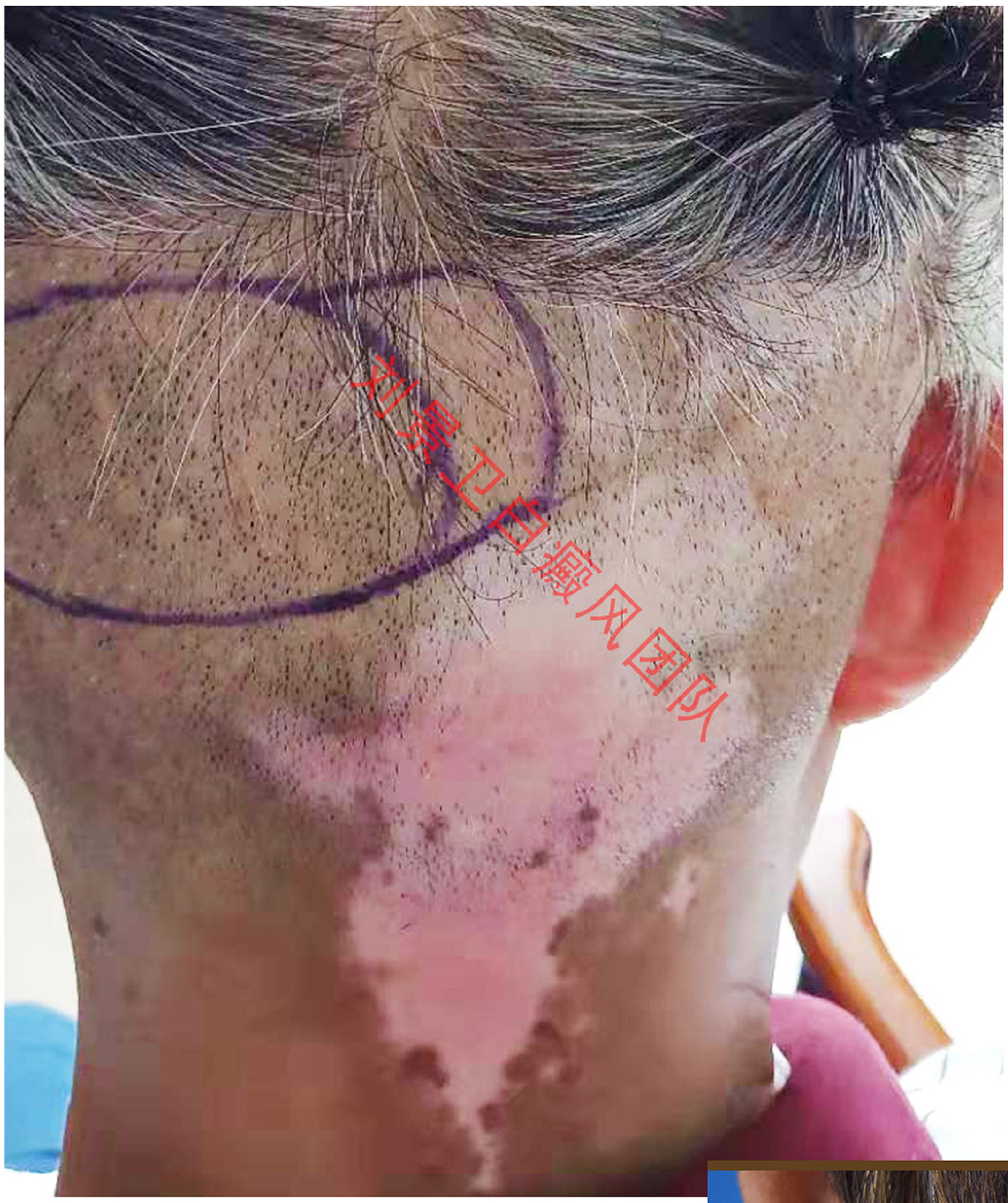

Before treatment

After treatment

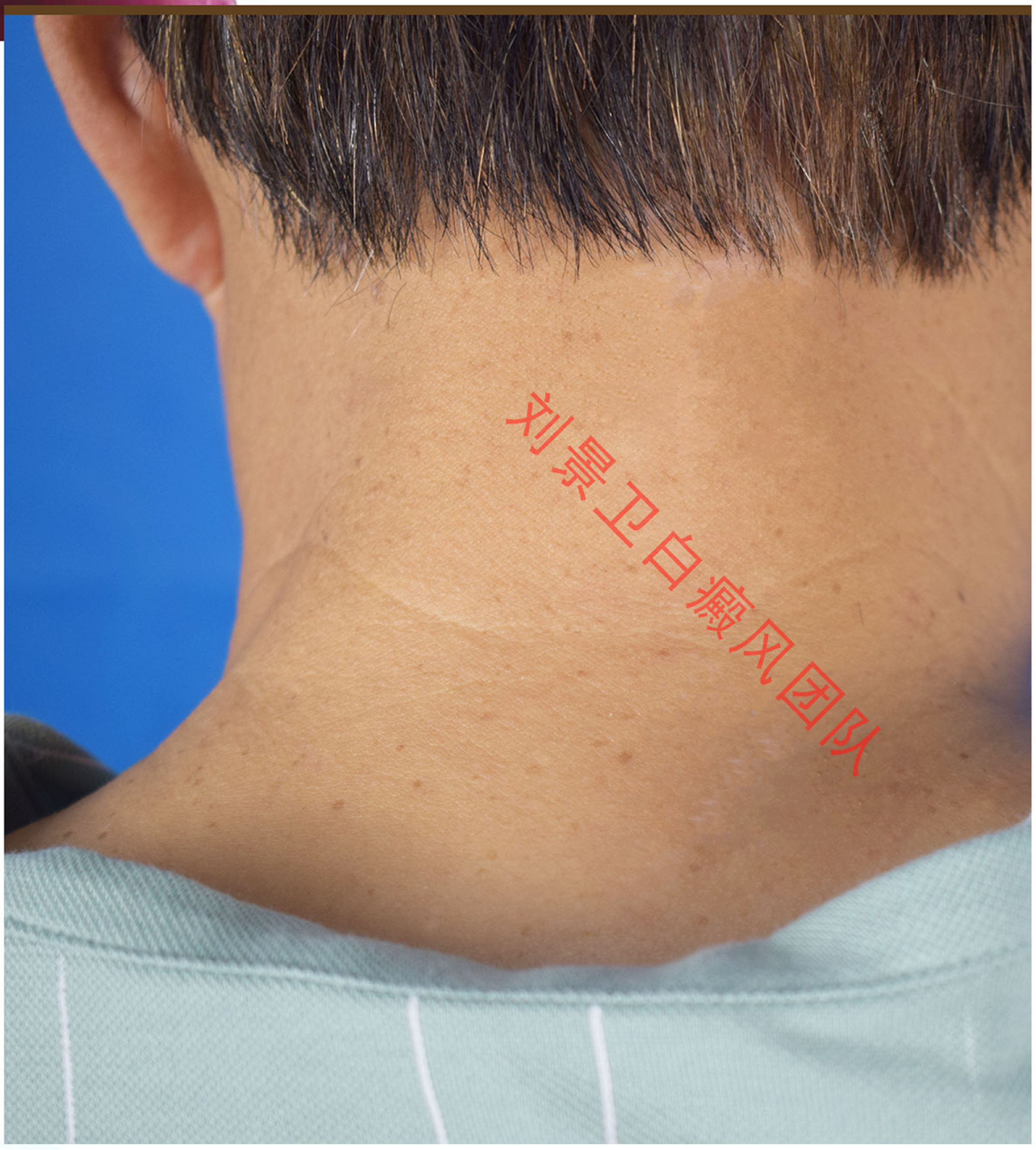

Cases of stem cell therapy for vitiligo

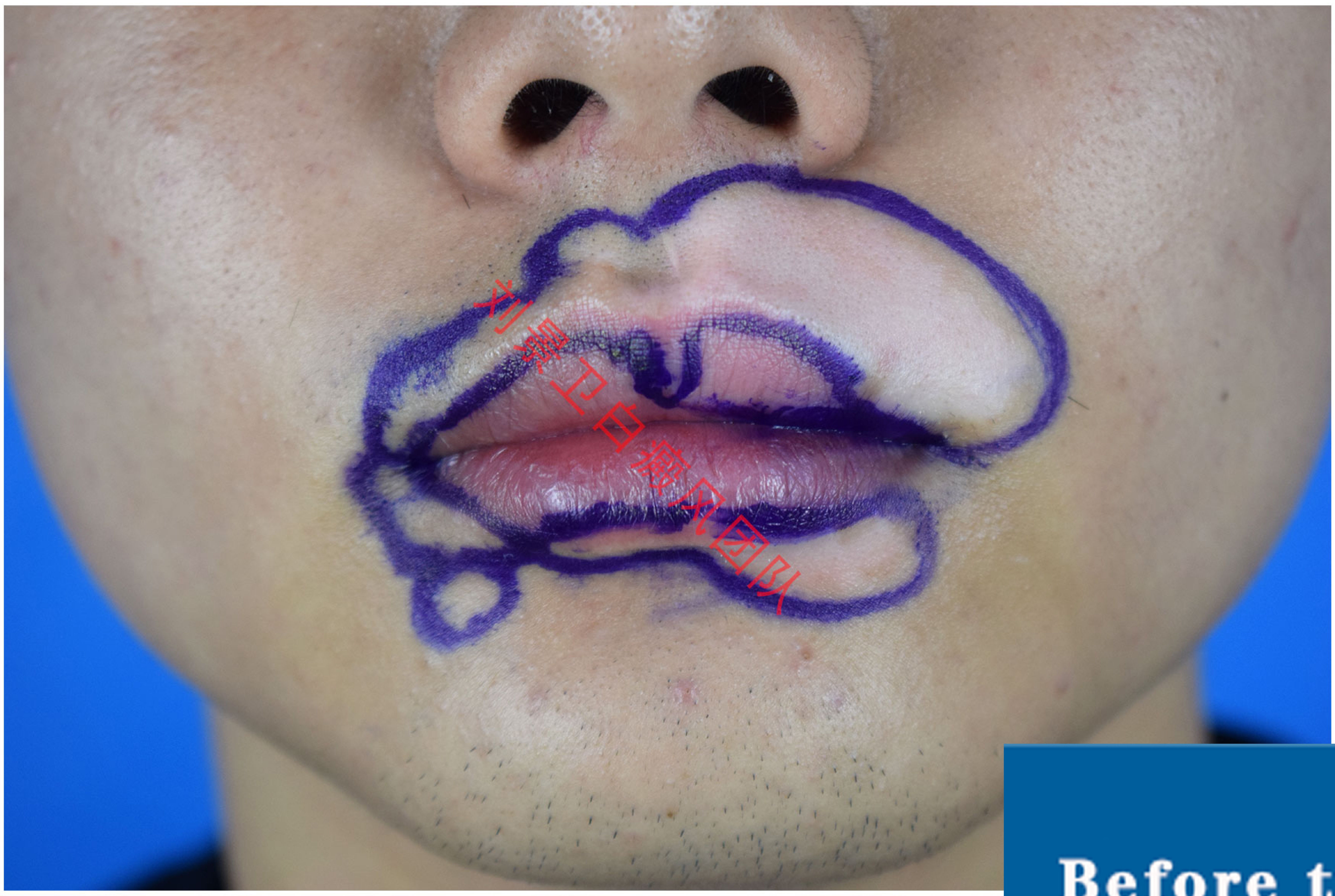

Before treatment

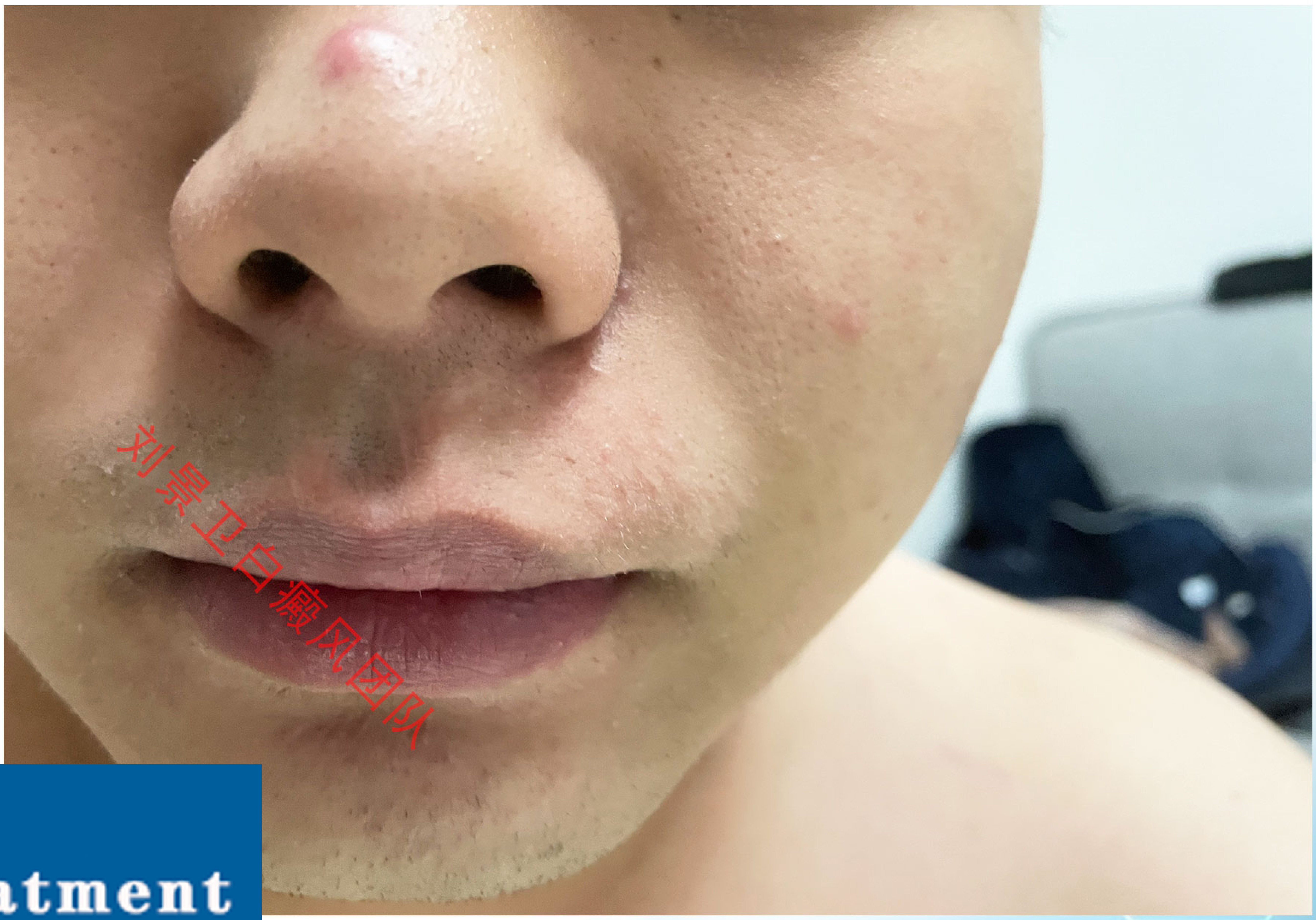

After treatment

Cases of stem cell therapy for vitiligo

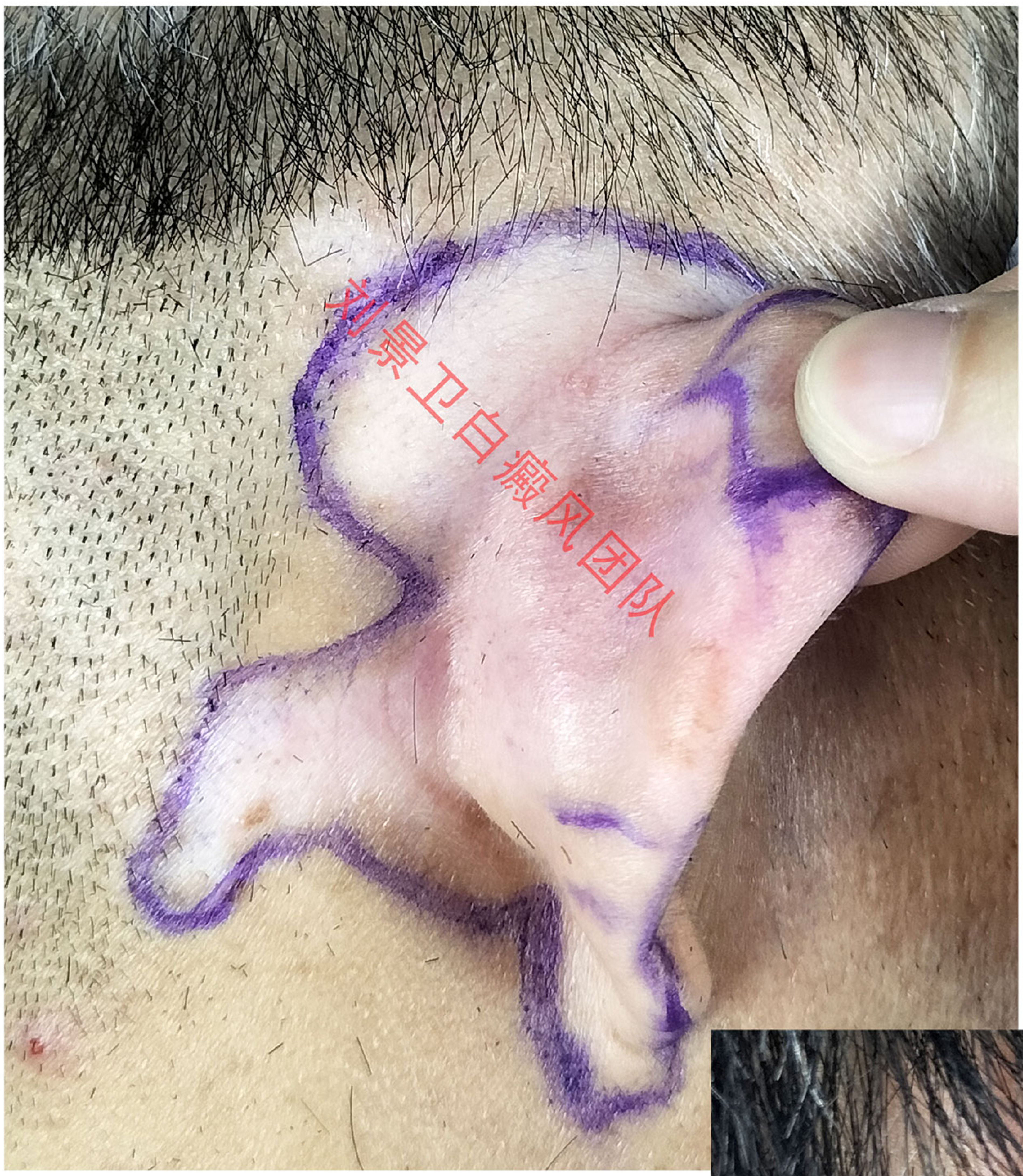

Before treatment

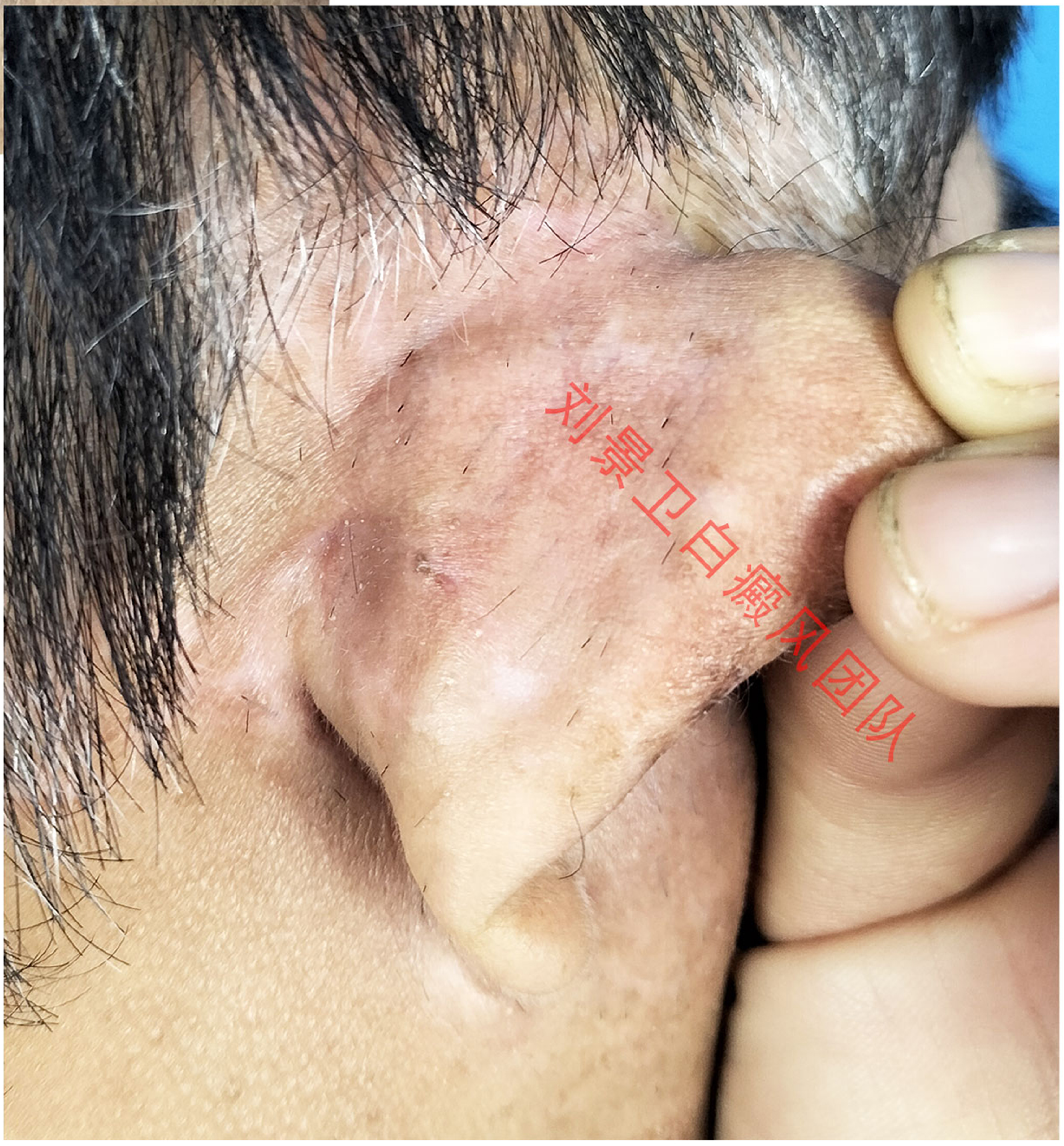

After treatment

Cases of stem cell therapy for vitiligo

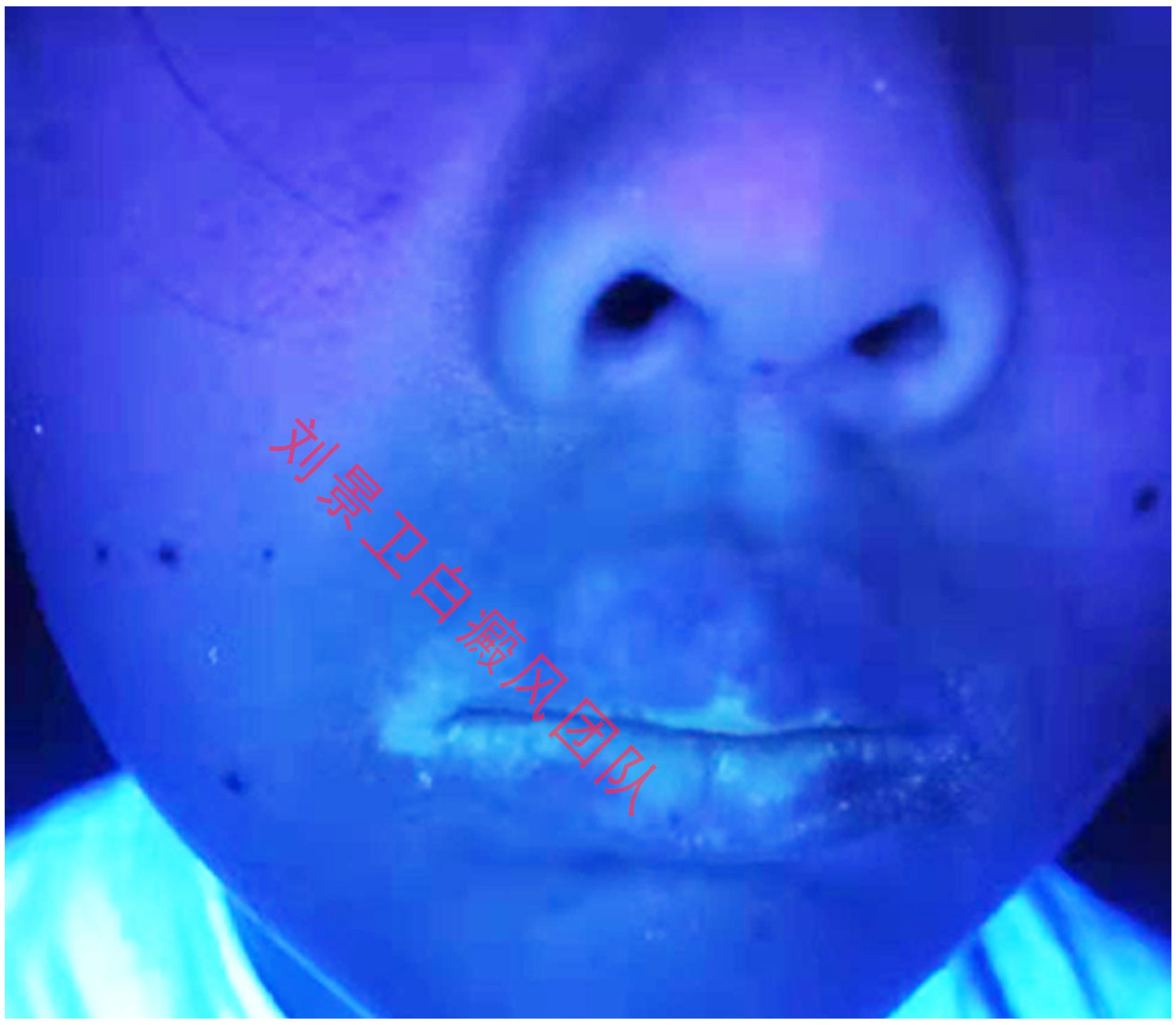
刘景卫白癜风团队

Before treatment

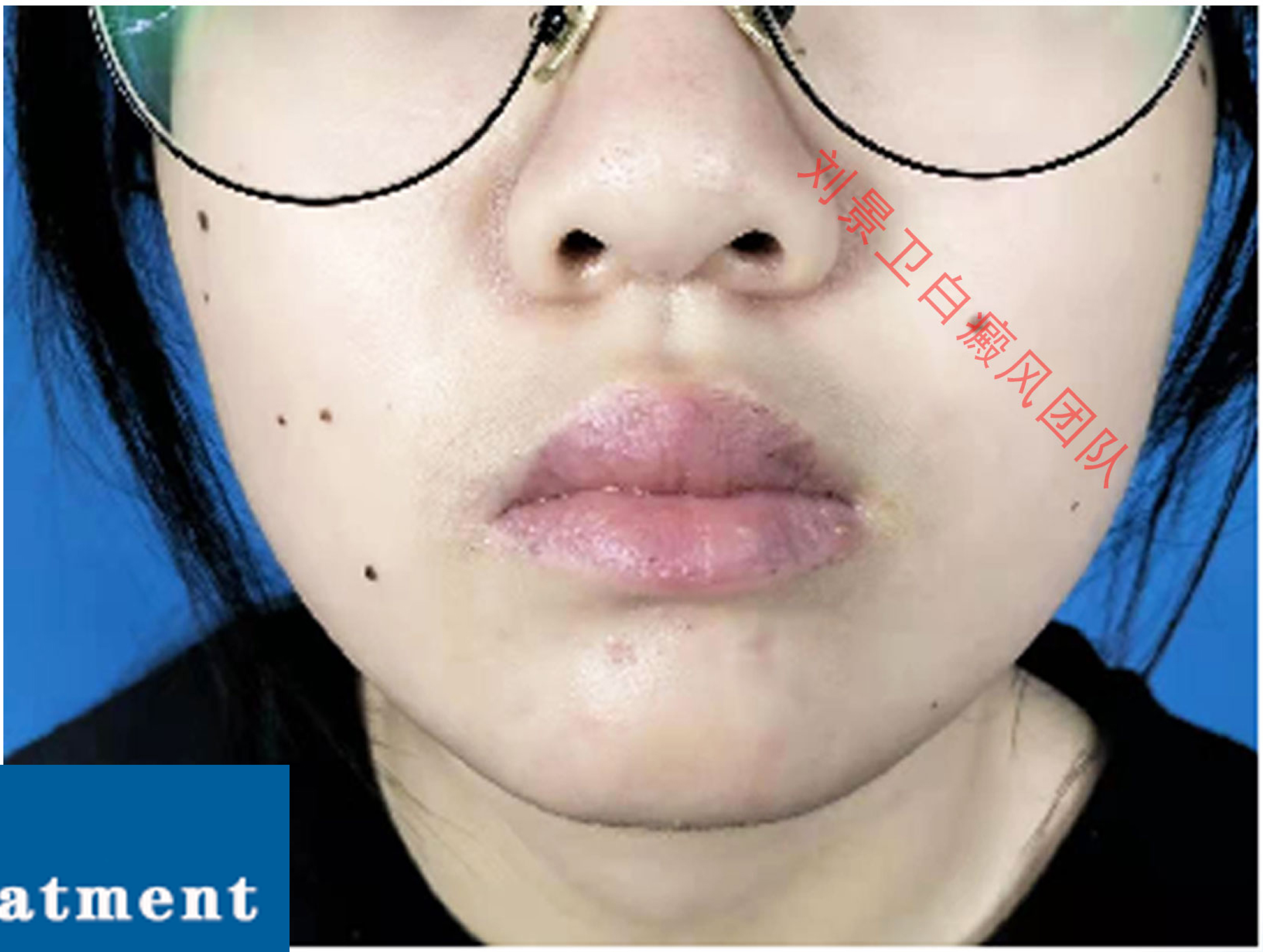
刘景卫白癜风团队

After treatment

Cases of stem cell therapy for vitiligo

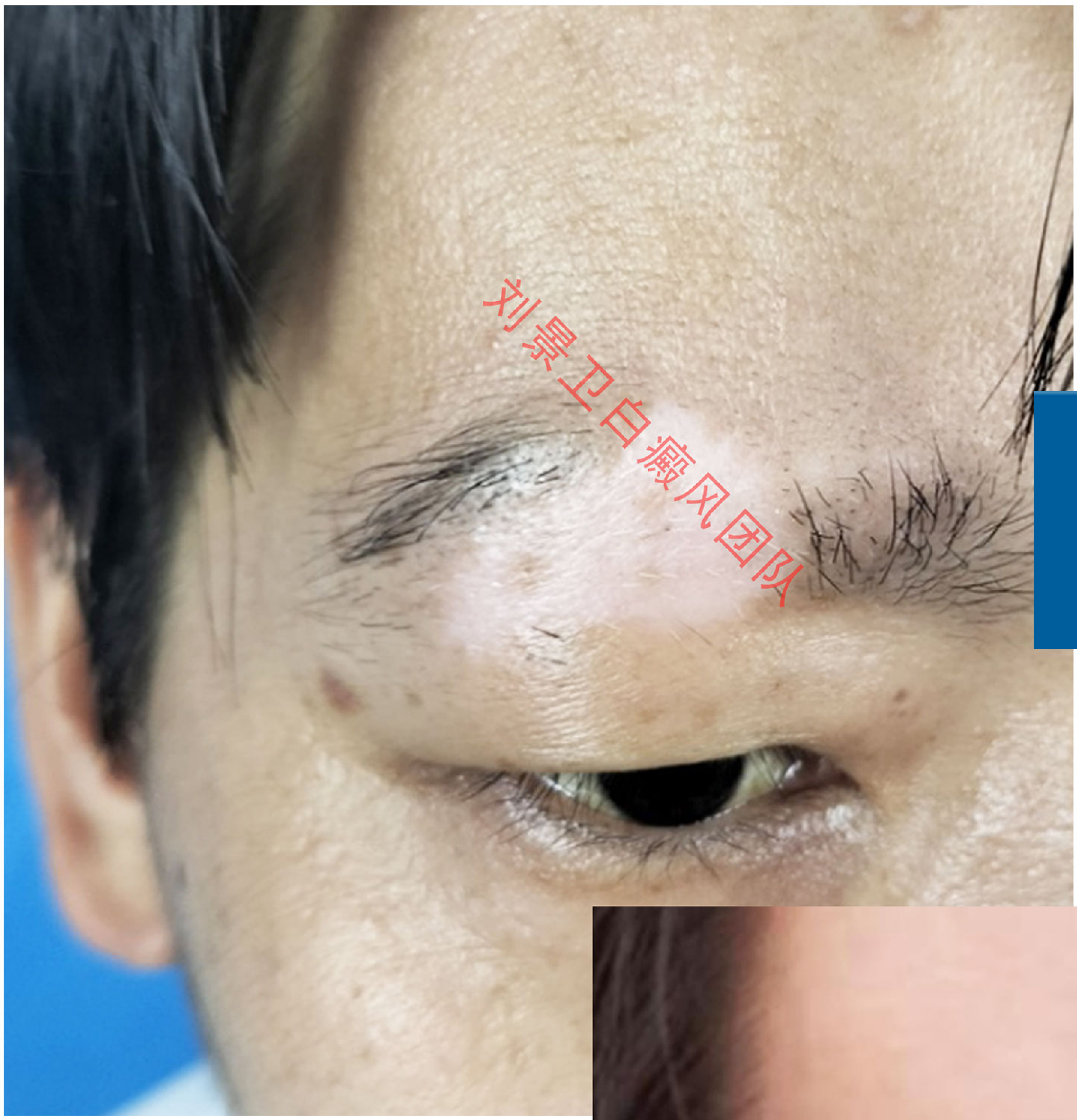

Before treatment

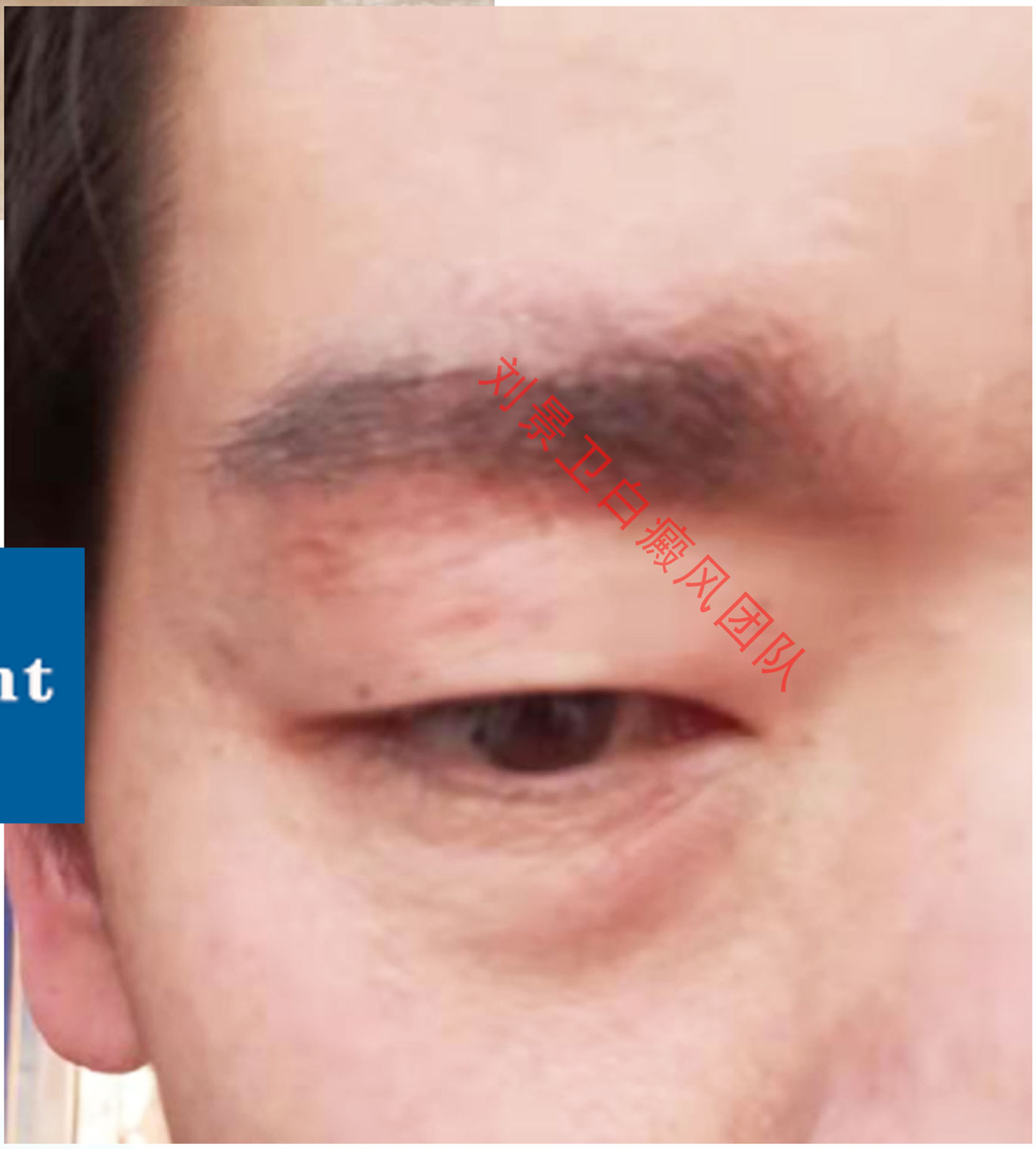

After treatment

Cases of stem cell therapy for vitiligo

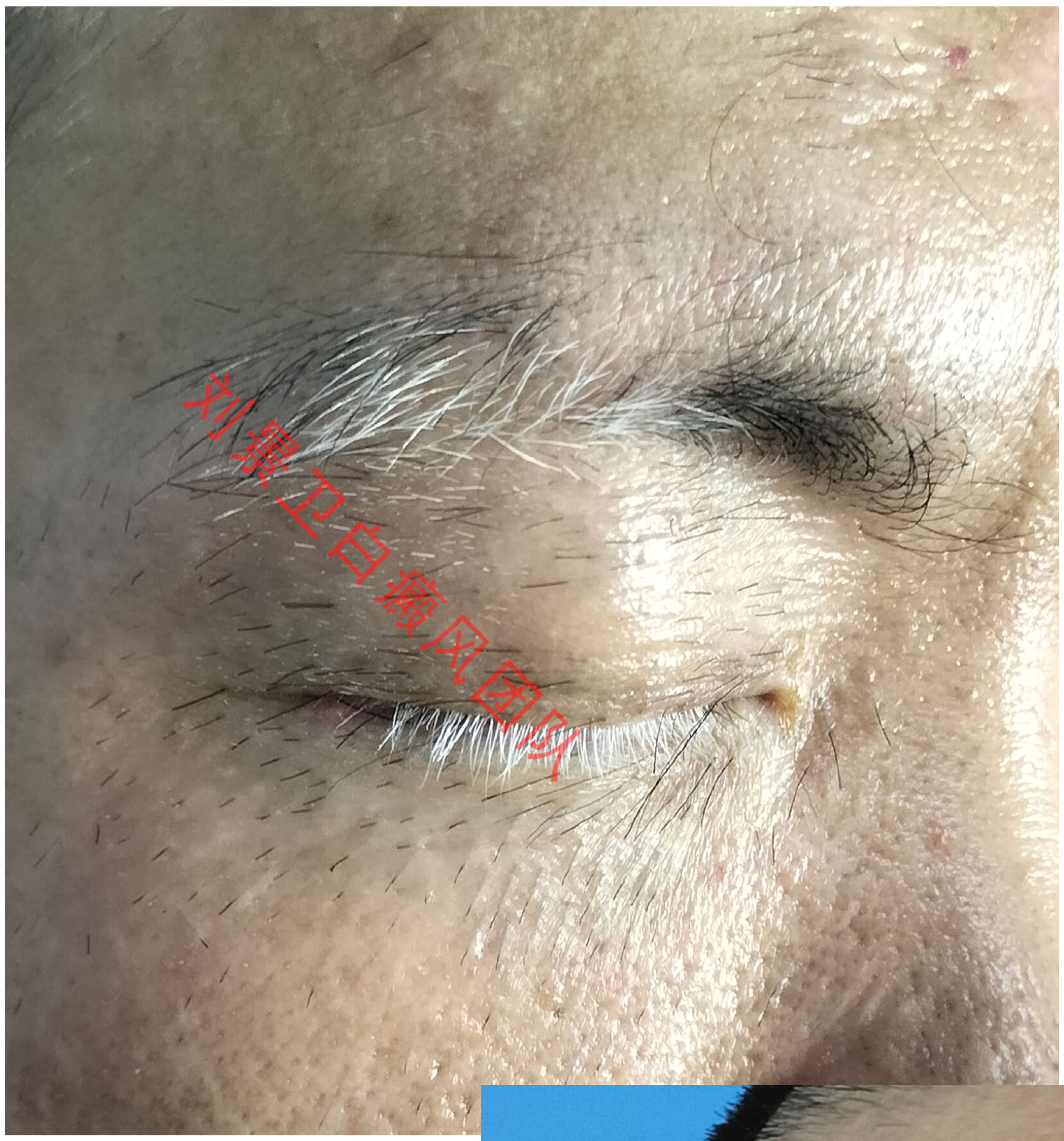

Before treatment

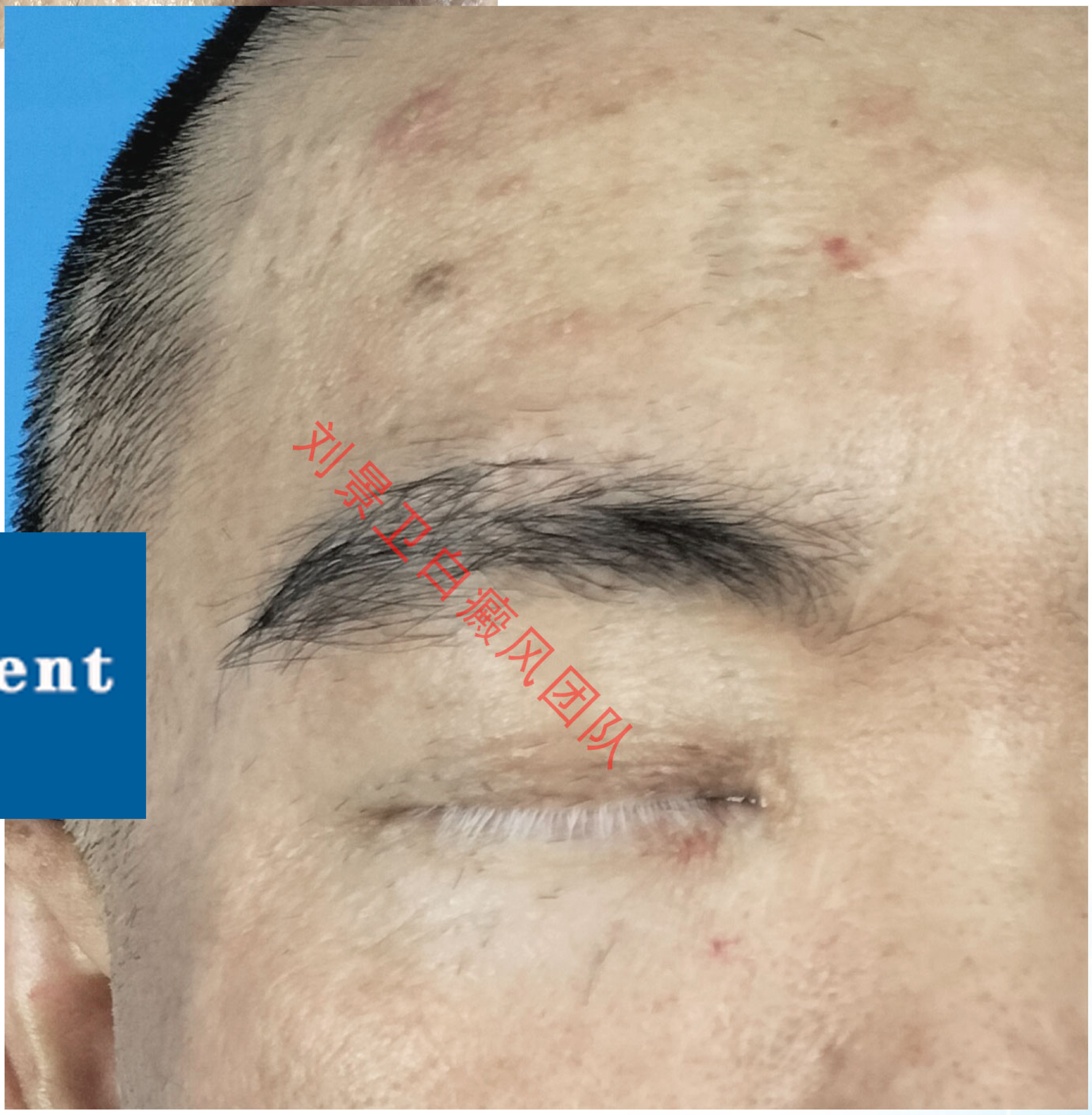

After treatment

Cases of stem cell therapy for vitiligo

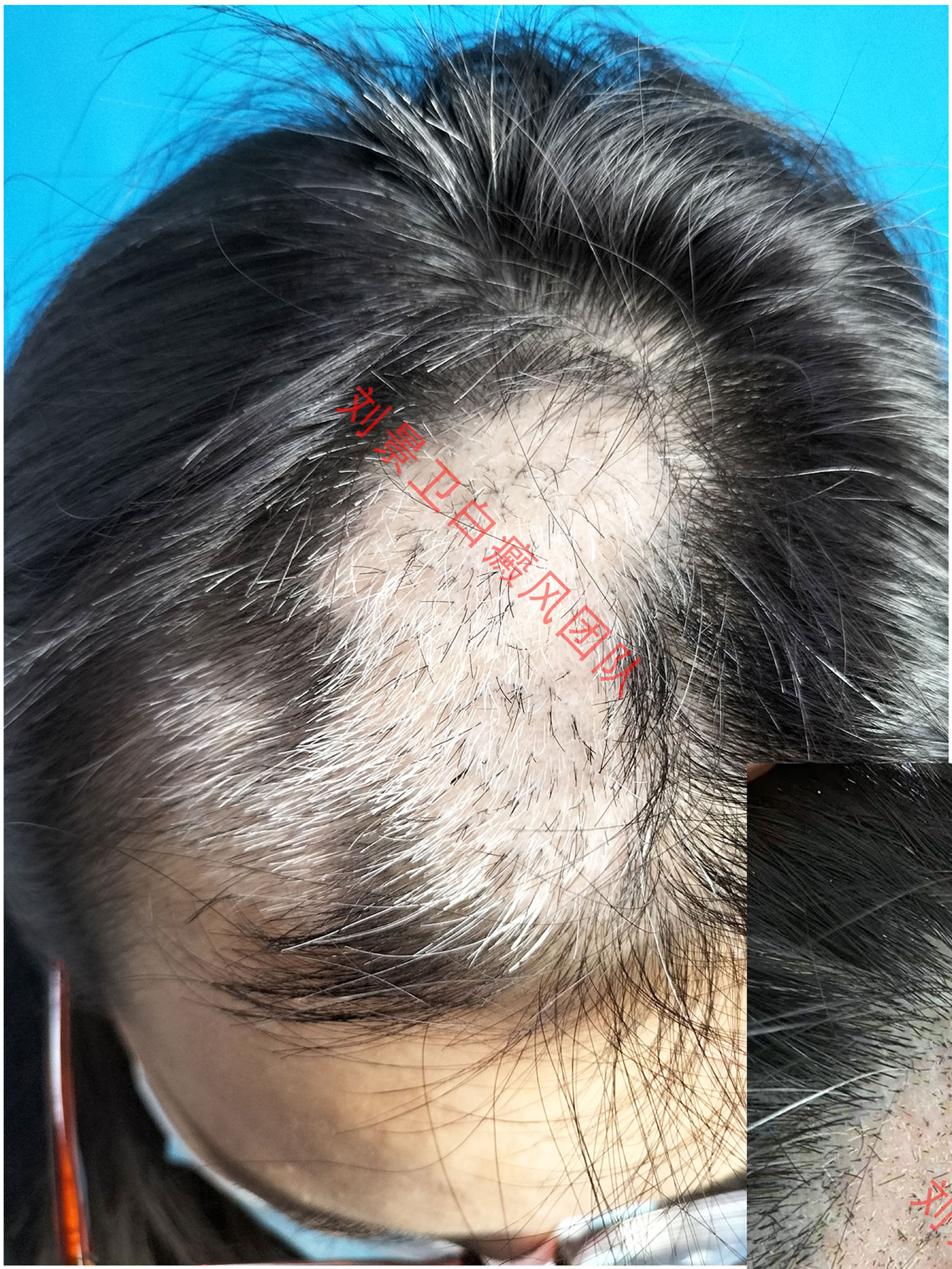

Before treatment

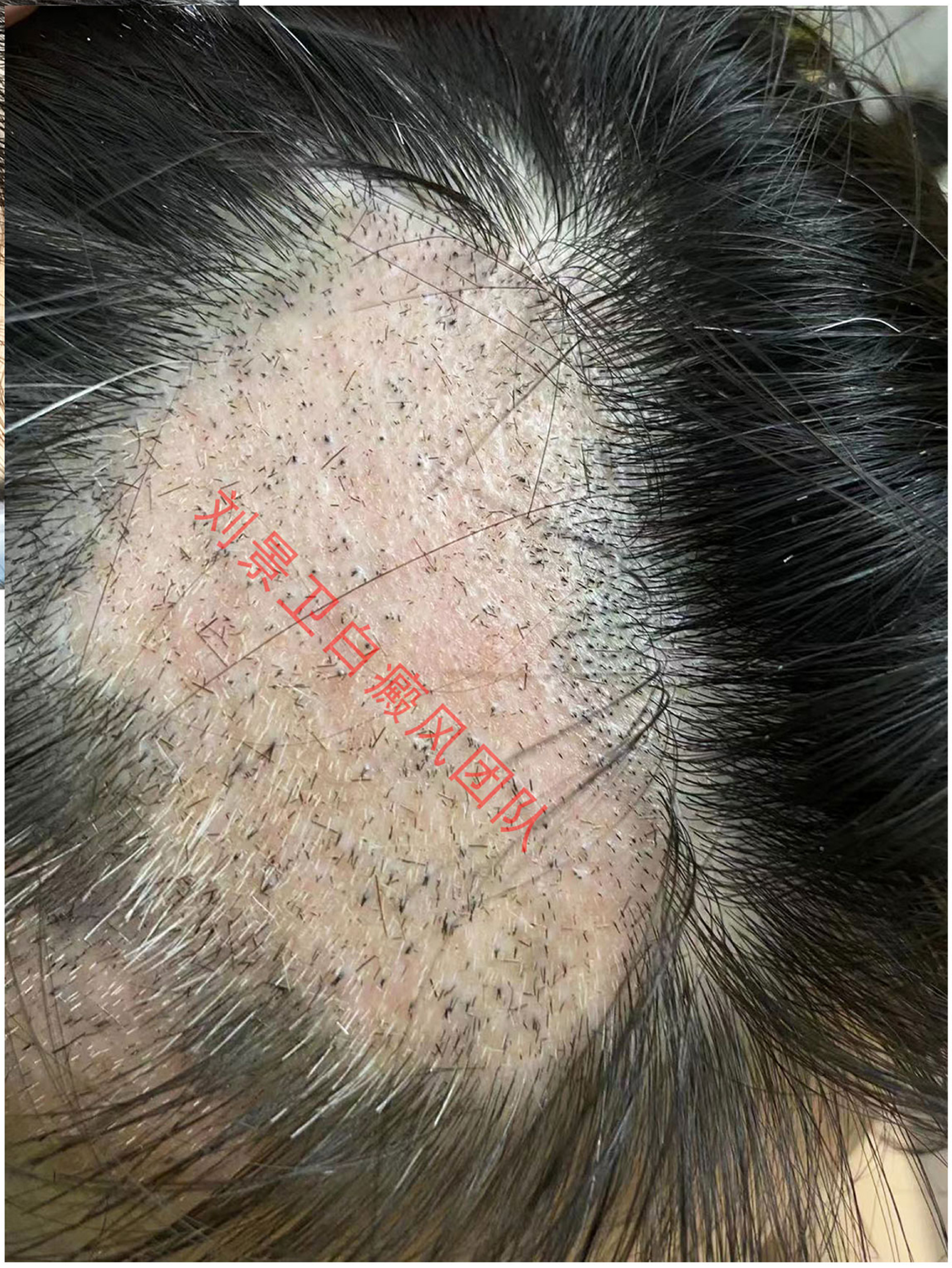

After treatment

Cases of stem cell therapy for vitiligo

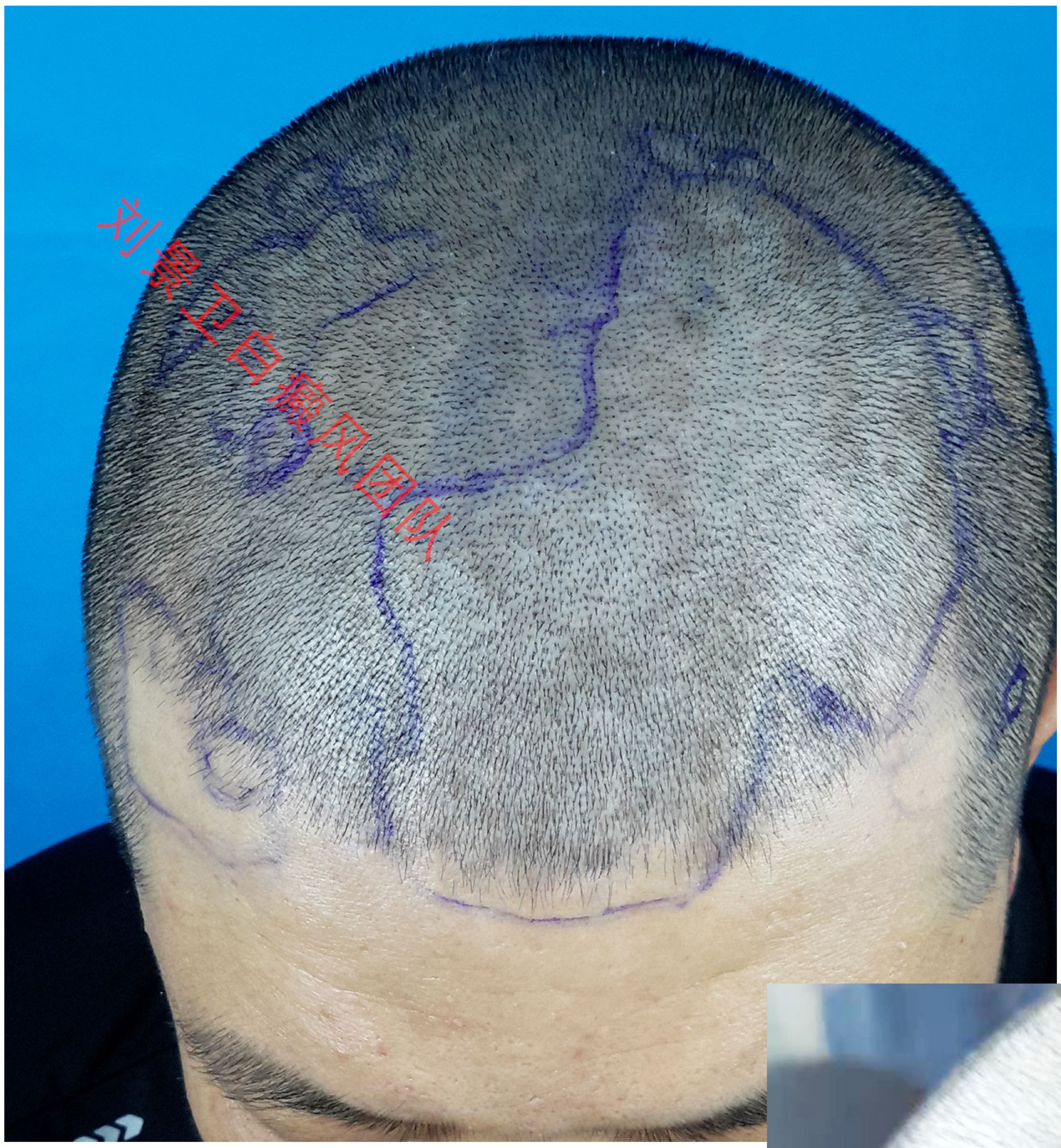

Before treatment

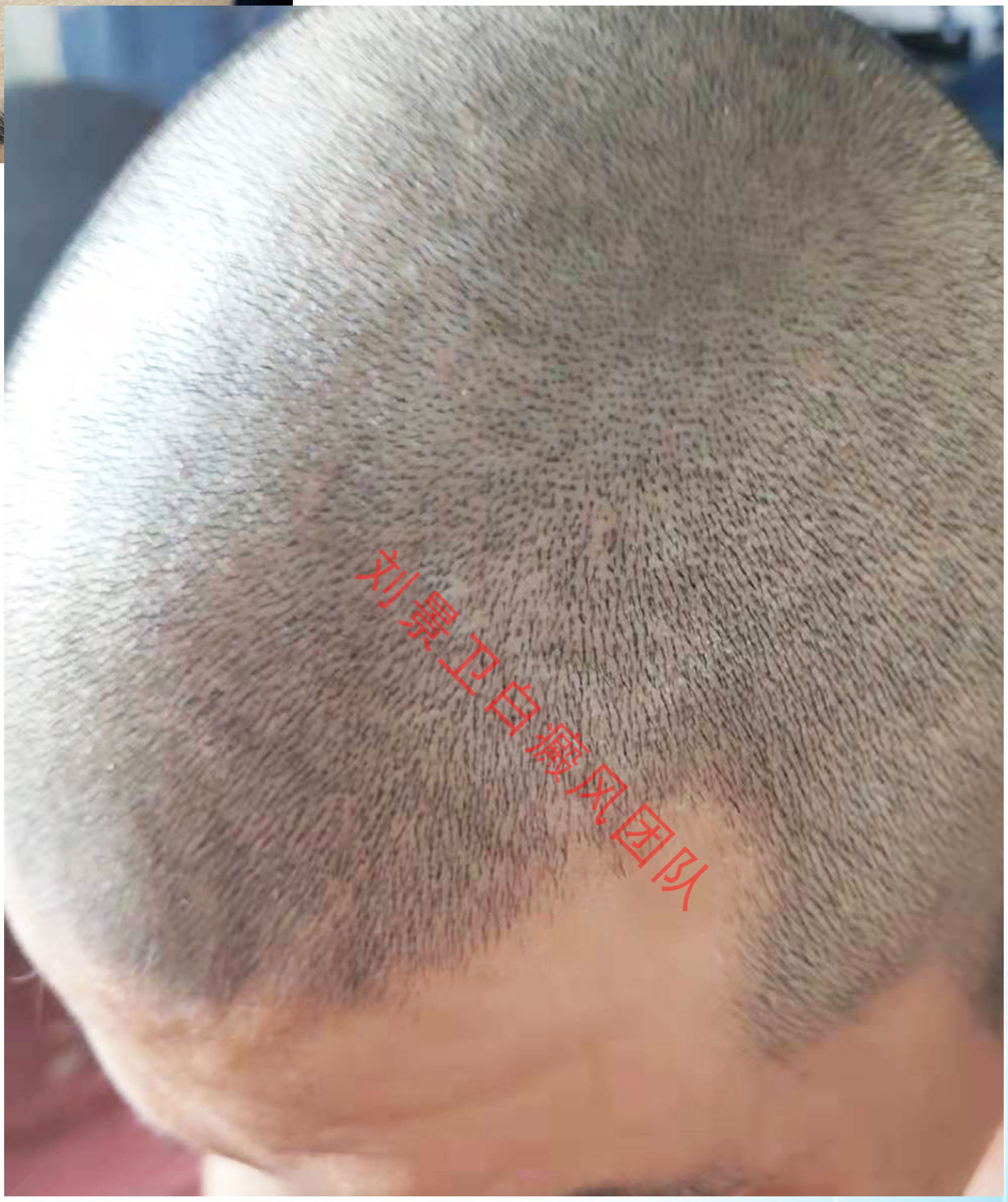

After treatment

Cases of stem cell therapy for vitiligo

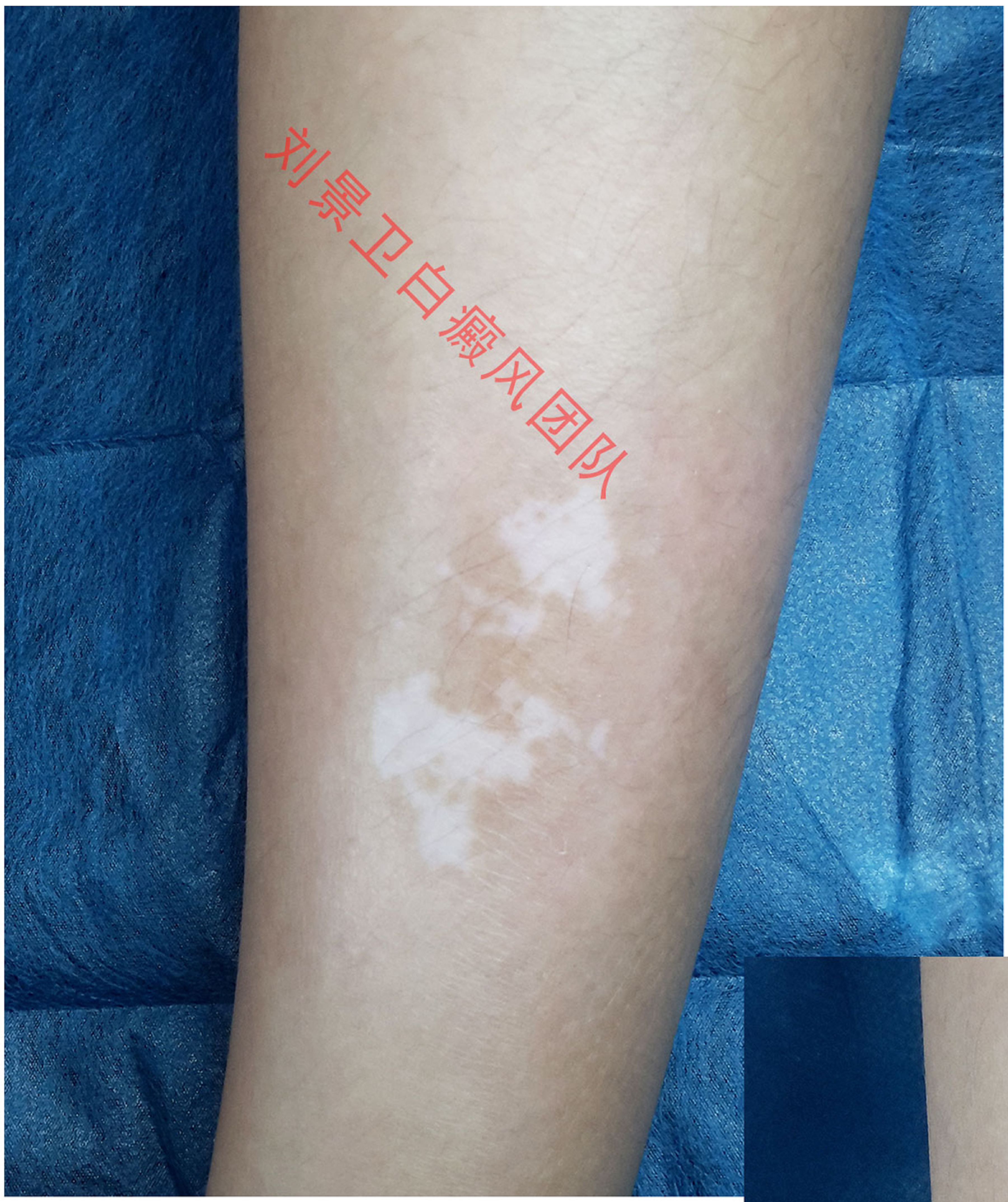

Before treatment

After treatment

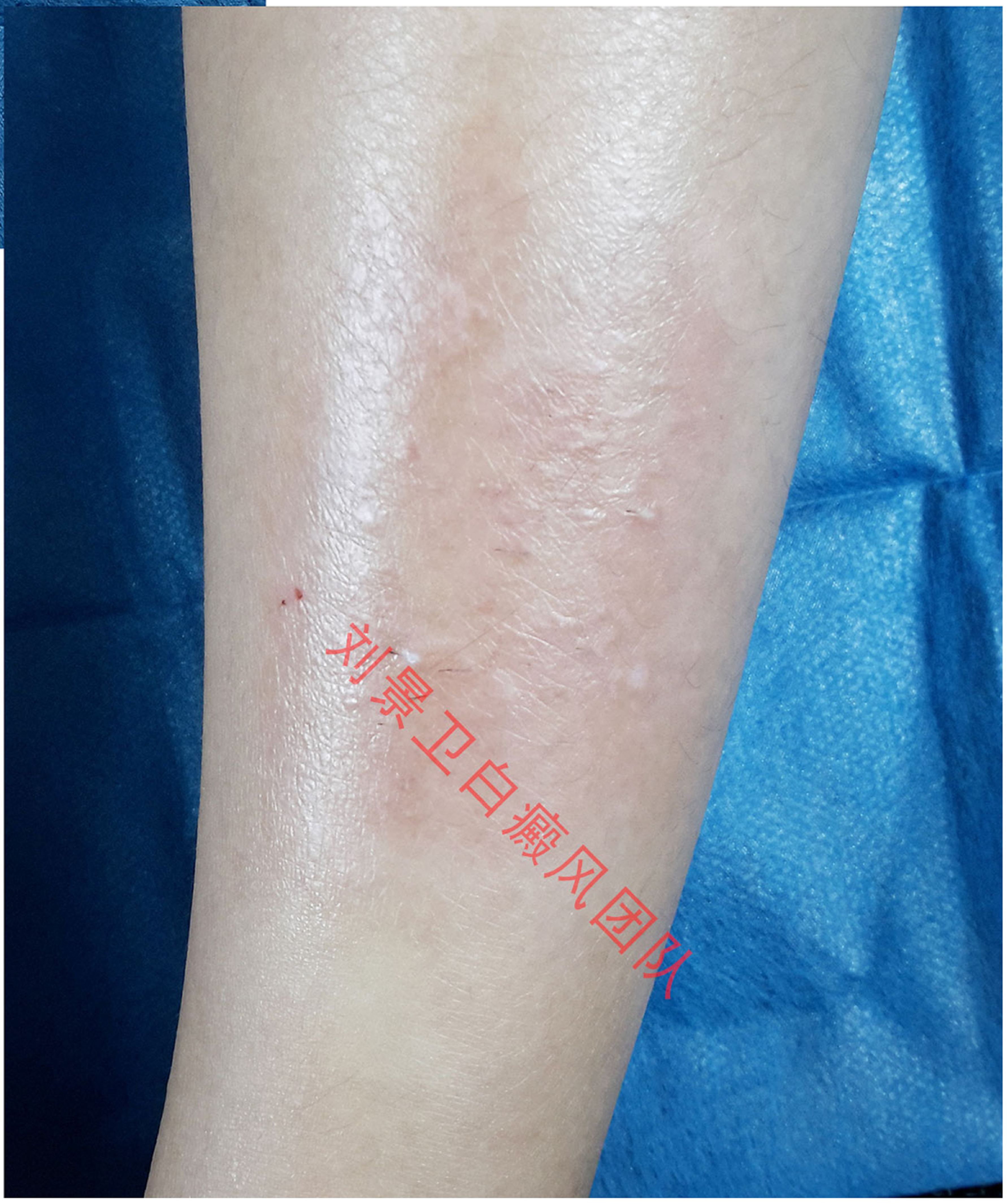

Cases of stem cell therapy for vitiligo

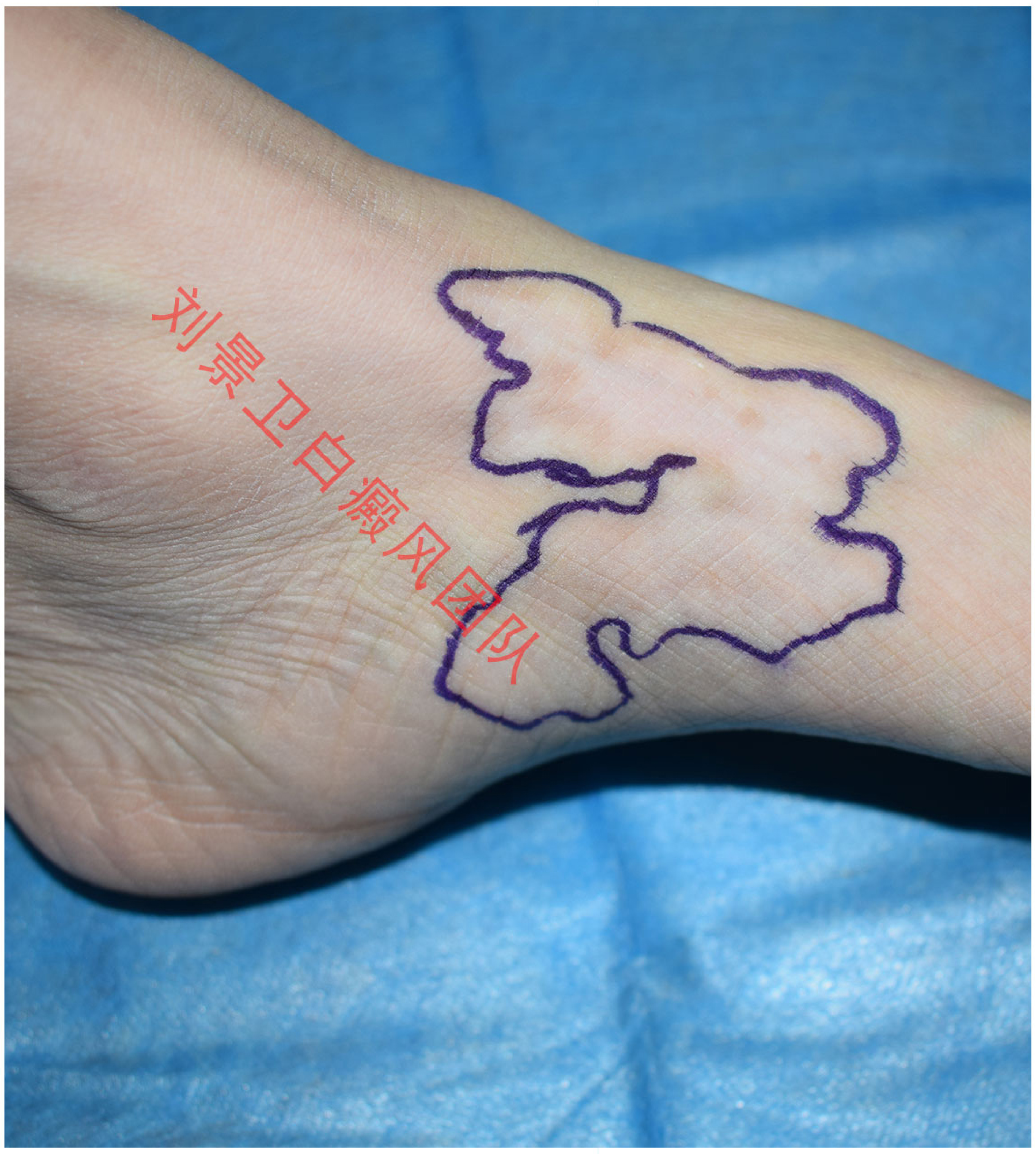

Before treatment

After treatment

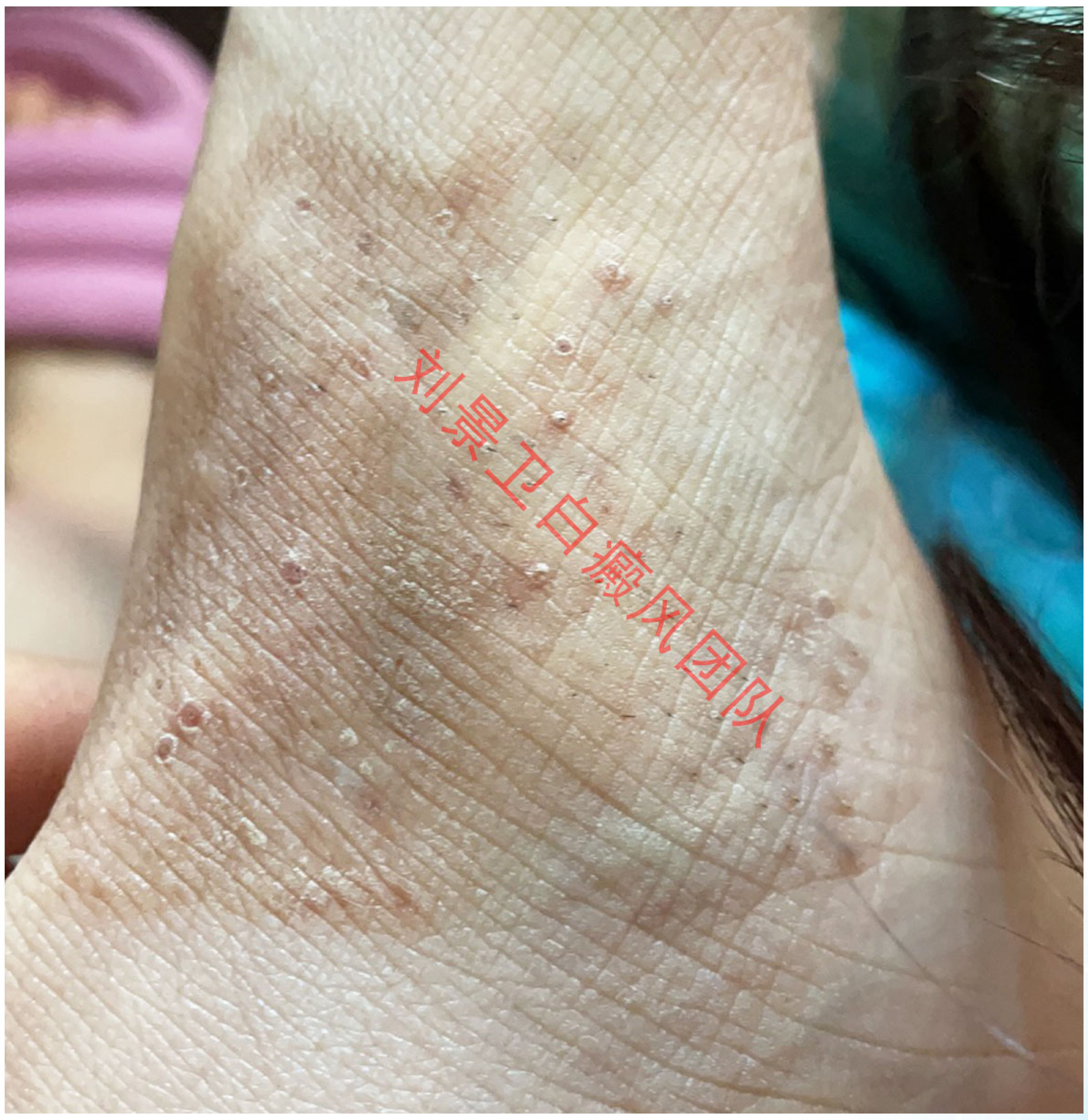

Cases of stem cell therapy for vitiligo

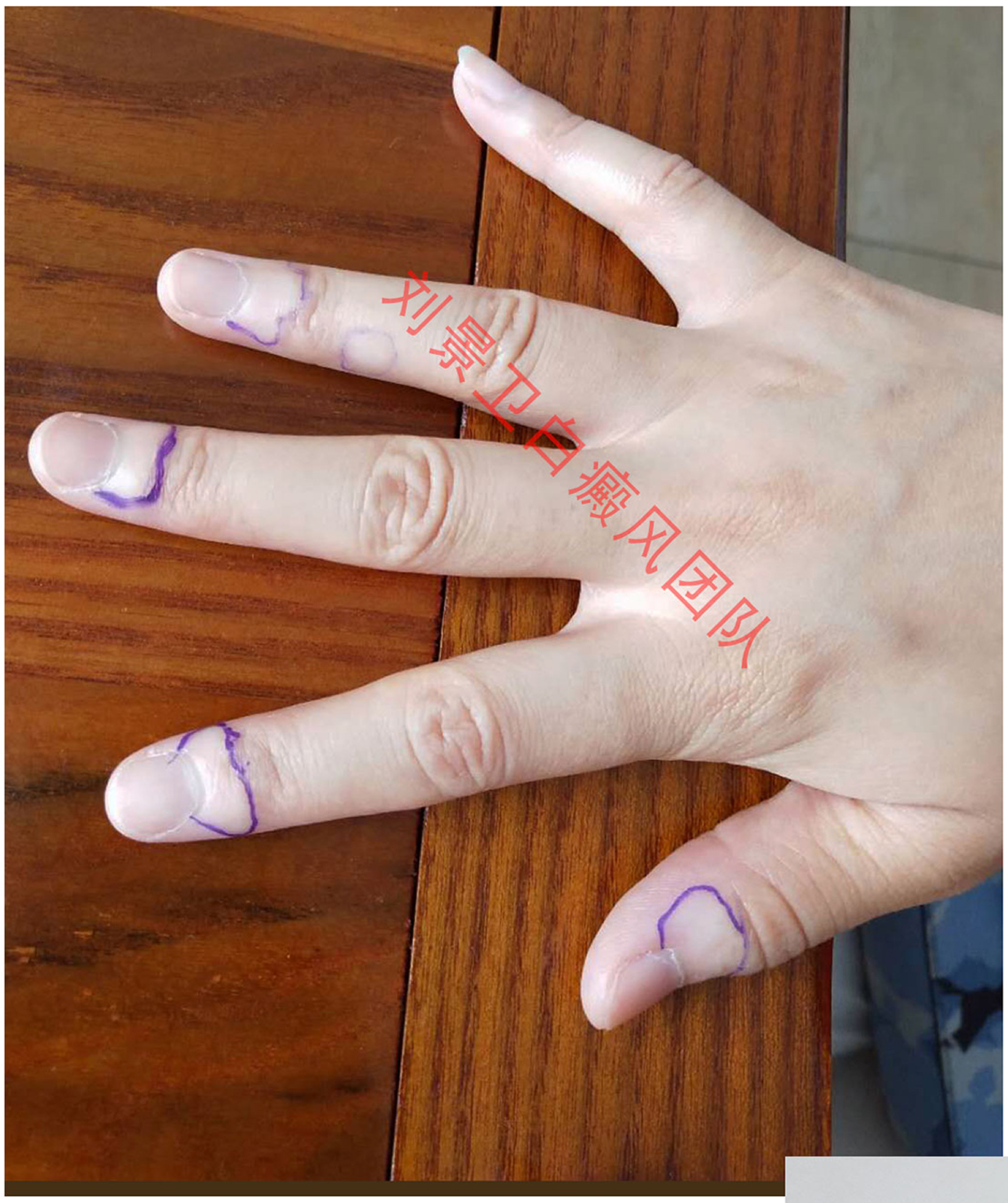

Before treatment

After treatment

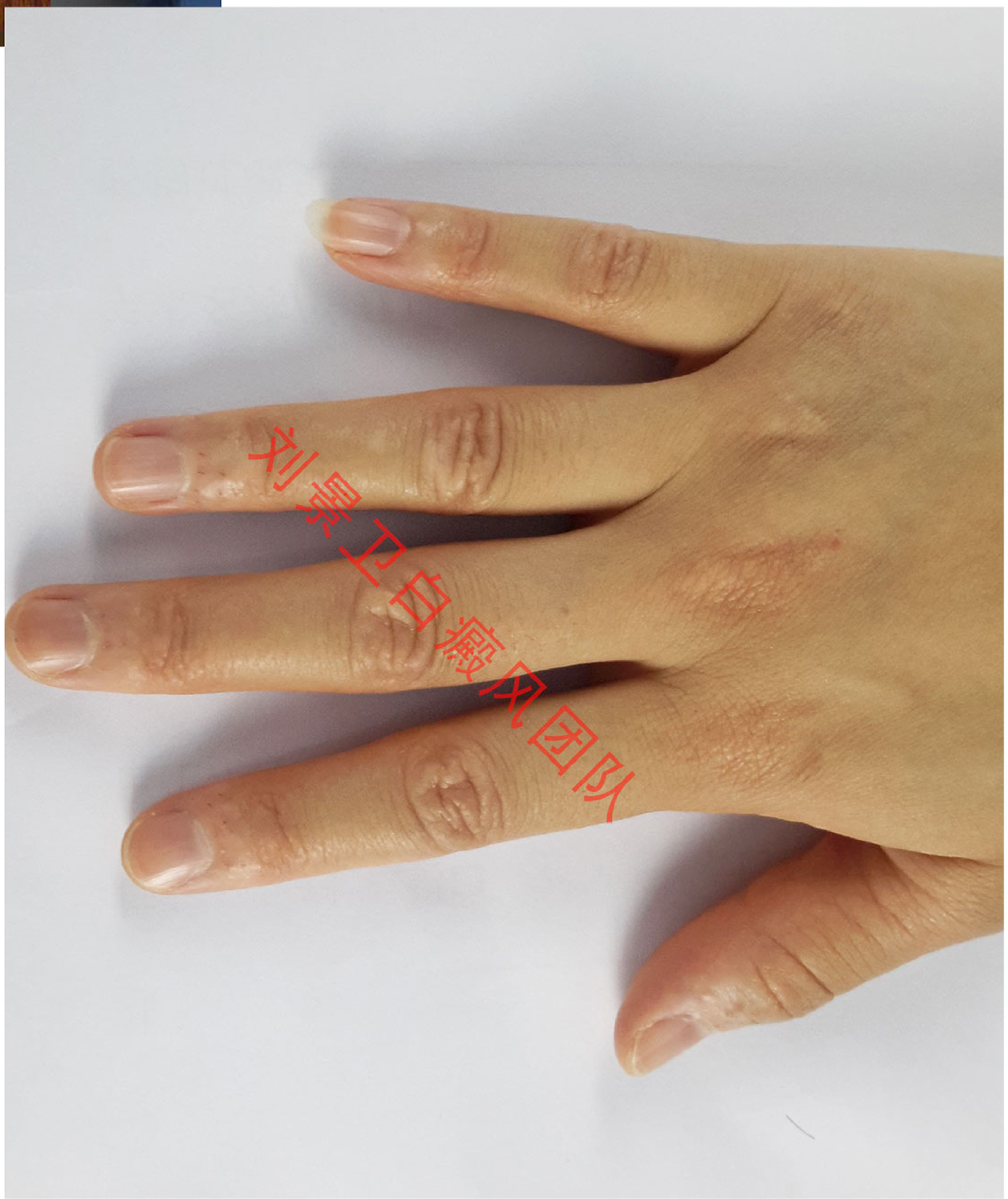

Cases of stem cell therapy for vitiligo

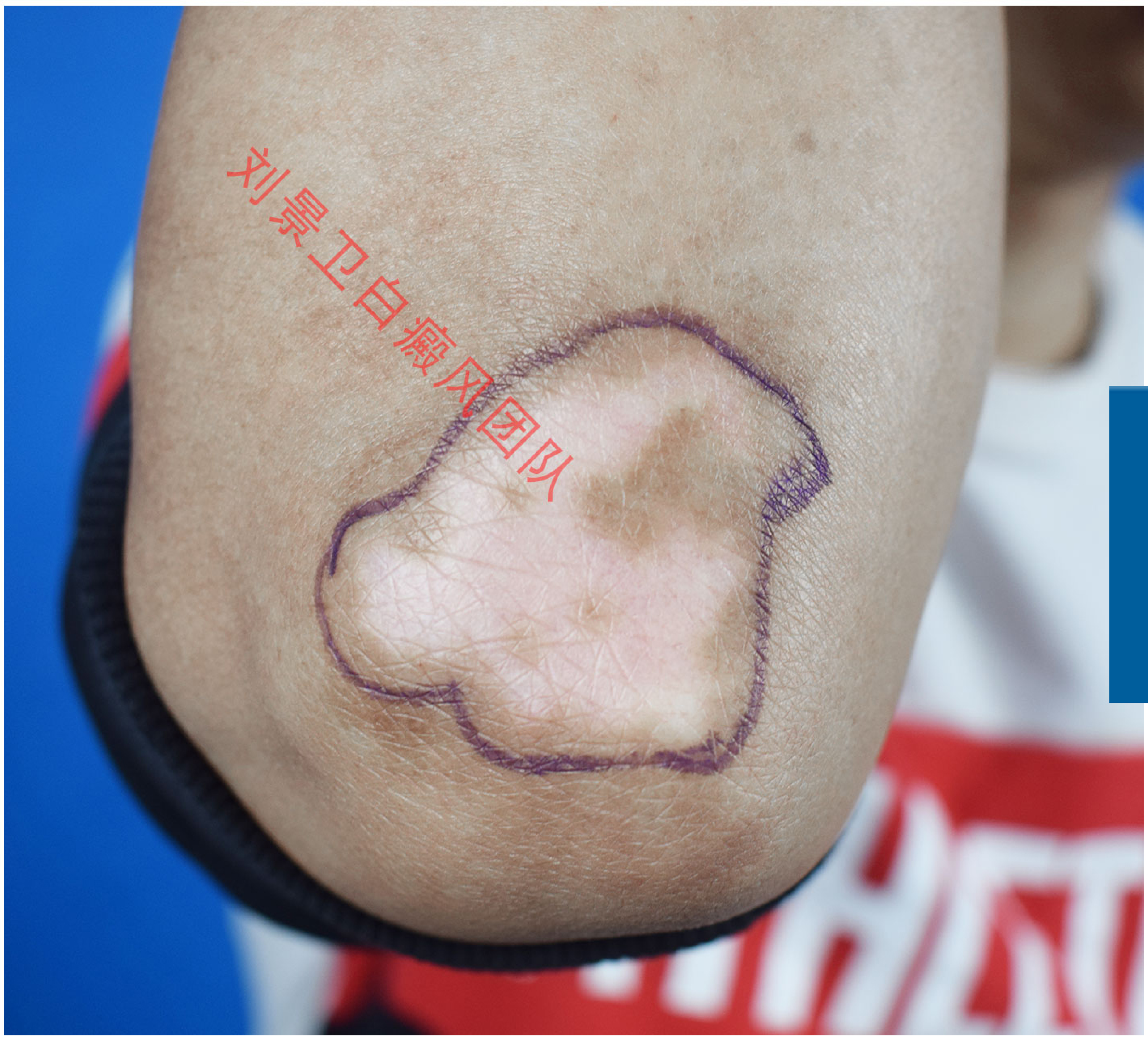

Before treatment

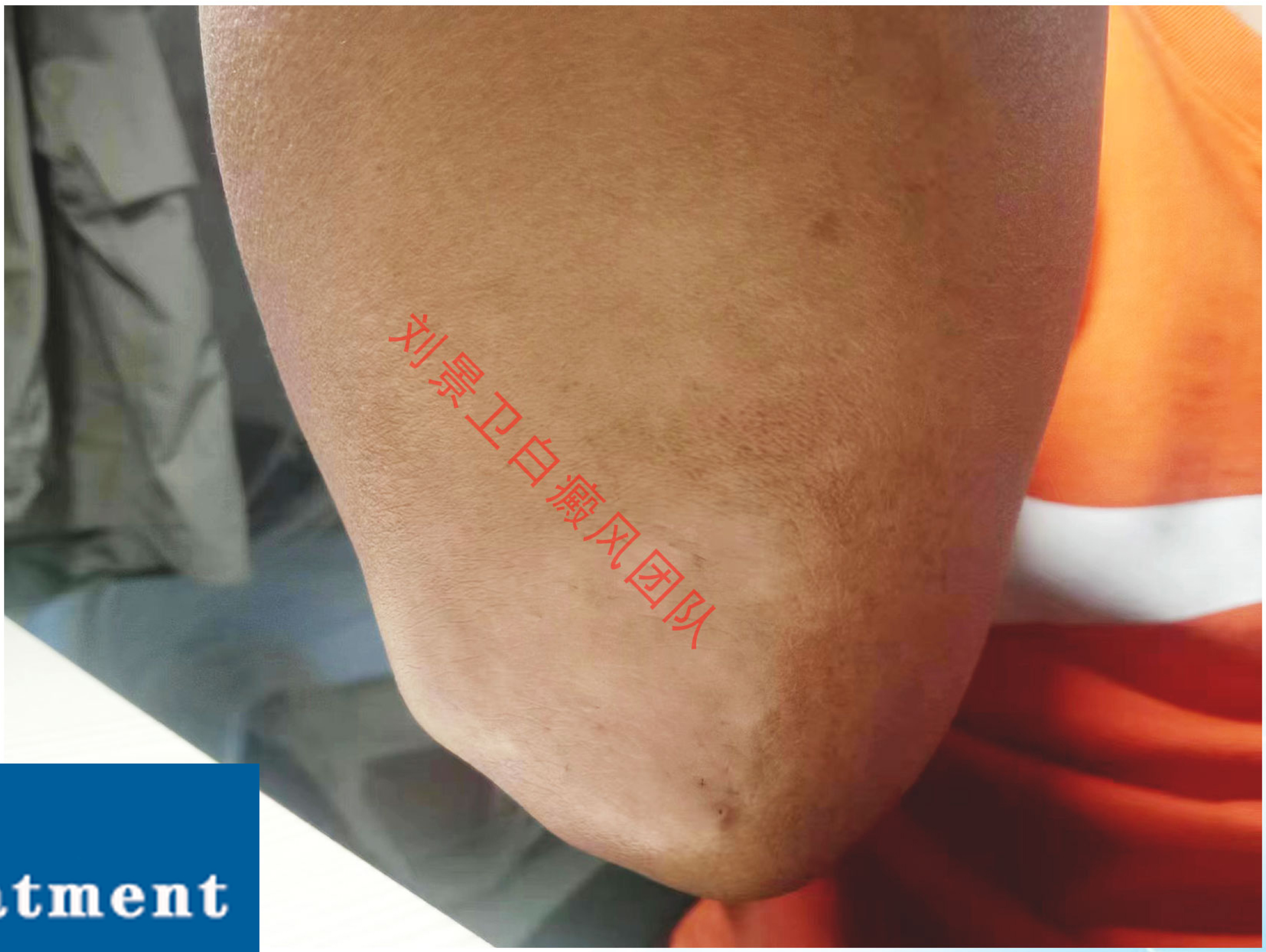

After treatment
