# Supplementary material for: Clinical validation and study of stem cell transplantation in treatment of vitiligo
Source: Arch Dermatol Res. 2023 Sep 7;315(10):2983–4. doi: 10.1007/s00403-023-02692-5 (PMC10615963; doi:10.1007/s00403-023-02692-5)
Supplement: Supplementary file 3 — Supplementary file3 (PDF 855 KB) [file 403_2023_2692_MOESM3_ESM.pdf]

海口仁术皮肤科门诊部有限公司伦理申请表

审查会议日期	2019 年 2 月 22 日
审查会议地点	南海仁术国际皮肤医院（海南）有限公司会议室
审查文件	完整的毛囊外毛根鞘移植治疗白癜风的临床研究与应用
临床研究单位	南海仁术国际皮肤医院（海南）有限公司
主要研究者	刘景卫、郭敏、陈青青、刘诗雨
伦理审查方式	流程讨论与现场手术临床观摩
参会委员	刘景卫、陈青青、陈春红、李道平、赵文志、陈克新、崔树成
审查意见	该疗法经审查委员会研究一致认为具有创新性、科学性、符合伦理学要求，可以开展该项目。
伦理委员会声明	该项目为解决世界性医学难题提供了一种疗效确切，可操作、符合伦理要求的新技术。
批件有效期	自 2019 年 2 月 22 日起<br>至 2025 年 4 月 21 日止
主任委员签字	
南海仁术国际皮肤医院（海南）有限公司伦理委员会（盖章）	
日期：2019 年 2 月 22 日	
