# Supplementary material for: Clinical validation and study of stem cell transplantation in treatment of vitiligo
Source: Arch Dermatol Res. 2023 Sep 7;315(10):2983–4. doi: 10.1007/s00403-023-02692-5 (PMC10615963; doi:10.1007/s00403-023-02692-5)
Supplement: Supplementary file 4 — Supplementary file4 (PDF 2109 KB) [file 403_2023_2692_MOESM4_ESM.pdf]

申报编号：SQ2021SHFZ0633

海南省重点研发计划任务书（2021年）

项目名称：完整的毛囊外毛根鞘移植术治疗白癜风的临床研究与应用

研究方向：社会发展

项目编号：ZDYF2021SHFZ048

承担单位：海口仁术皮肤科门诊部有限公司

项目负责人：刘景卫　联系电话：089866266732　手机：13368984595

项目联系人：刘景卫　联系电话：13368984595　手机：13368984595

起止时间：2021年09月—2023年09月

海南省科学技术厅

二〇二一年

海南省科技计划项目任务书签批审核表

项目负责人填写

我同意承担海南省科技计划项目，将按照申请书、项目立项通知和任务书负责实施本项目（项目编号：ZDYF2021SHFZ048），严格遵守海南省科技厅关于项目管理、财务管理等各项规定，切实保证研究工作时间，认真开展研究工作，按时报送有关材料，及时报告重大情况变动，对资助项目发表的论著和取得的研究成果标注“海南省科技项目资助”字样及项目编号。

项目负责人（签章）：[illegible]

2021 年12月13日

承担单位填写

我单位同意承担上述海南省科技计划项目，将履行有关承诺，保证项目负责人及其研究队伍的稳定和研究项目实施所需的条件，严格遵守海南省科技厅有关科技项目管理、财务管理等各项规定，并督促实施。

承担单位（公章）

2021 年12月13日

参加单位填写

我单位同意参加上述海南省科技计划项目，将履行有关承诺，提供相关配合，协助项目承担单位和负责人完成项目实施。

参加单位（公章）

年 月 日

我单位同意参加上述海南省科技计划项目，将履行有关承诺，提供相关配合，协助项目承担单位和负责人完成项目实施。

参加单位（公章）

年 月 日

省科技厅填写

同意按计划执行

（盖章）：

2021年 11月 9日

分管处室：	社会发展科技处	联系人：	刘建勋	联系电话：	66290357
