# Supplementary material for: Clinical validation and study of stem cell transplantation in treatment of vitiligo
Source: Arch Dermatol Res. 2023 Sep 7;315(10):2983–4. doi: 10.1007/s00403-023-02692-5 (PMC10615963; doi:10.1007/s00403-023-02692-5)
Supplement: Supplementary file 5 — Supplementary file5 (PDF 1563 KB) [file 403_2023_2692_MOESM5_ESM.pdf]

毛囊黑素细胞干细胞移植术治疗白癜风知情同意书

患者	郭义鹏	性别	男	年龄	30 岁	部位	面部
单位地址							
诊断	面部白癜风			手术日期	2020.7.3		
手术名称	毛囊黑色素移植术			电话	15120608372		
身份证编号	460022199010060715	如不愿提供身份证号请签字确认					

※专家帮我设计的手术方案，我已满意，签名 郭义鹏

1. 患者 郭义鹏 知道并了解医师 刘景卫 及其白癜风手术小组准备为我进行白癜风手术。我也知道手术中包括的其他一些必要且合理的医疗服务，如麻醉剂和镇静剂的使用等。
2. 我知道理想的手术效果一定程度上取决于全部完成医师推荐的手术。但是，因为许多因素是不确定的，因此我知道并不是通过手术就一定能够取得预期的效果。我也知道现有的毛发数量和质量是决定最终手术效果的主要因素。
3. 在知情同意之前，我确定已经阅读或已经清楚下述事项。
   手术并发症、手术前和手术后的事项及用药、手术的费用
4. 我完全知道自己预期的合理目标。我知道白癜风手术不是十全十美.医师已经向我解释了手术的过程，我也有机会向医师询问有关手术的问题。我也知道手术后的许多天内都可以明显地看到受区位点，手术治愈率只有 90%左右，医生会努力帮我达到最好疗效。手术只能治疗白癜风，不能预防白癜风。
5. 医师建议我最少需要一次手术。我知道在咨询和治疗过程中医师给予的建议也只是估计，以后还是可以改变的。如果医师或者我自己觉得有必要增加另外的治疗，我知道治疗的费用是额外的。
6. 医师已经告诉我毛囊黑素细胞干细胞移植是相对比较安全的手术，但我知道也有可能发生一些并发症，医师已经向我交代了最为常见的以及一些少见的并发症，而且我已经阅读了知情同意书中列出的并发症，包括：药物和麻醉剂的意外反应、不常见的感染和少见的伤口愈合反应等。虽然医师不能仔细地向我解释每一种都可能会发生的并发症，但我知道可能会发生的一切风险。
7. 我知道并同意由 刘景卫 及其助手为我进行手术，我也知道所使用的毛囊黑素细胞干细胞移植术。

南海仁术国际皮肤 [illegible]
46010100291[illegible]

8. 我确定已经知情同意。我知道可以预期理想的手术效果，但是使用的手术与药物并不一定是完全科学的。我知道经验丰富的医师有时候即使使用最好的方法也不一定就能取得理想的结果。
9. 我知道毛囊黑素细胞干细胞移植手术的成功与否取决于我是否遵循了医嘱，这包括（但也并不局限于）：术前术后的注意事项，医师已经向我交代清楚。我同时会全力配合医生给我的治疗方案，我阅读了知情同意书并进行签名，而且在这过程中我没有服用任何能够影响我思维的药物。
10. 我确定我已经阅读了知情同意书，或者医师向我阅读了知情同意书，而且已经填写了空白的内容，我知道所填写的内容。
11. 我已经告诉医师自己既往和现在的健康状况，目前药物使用情况以及已知的药物过敏史。这些信息是非常重要的，因为这可以指导医师在毛囊黑素细胞干细胞移植的过程中采取正确的处理方法。
12. 术后可能会有一些不舒适的感觉，如果术后有毛发生长，医生会为我免费处理。提供本知情同意书完全是为了取得最好的手术效果，并没有任何其他利益的动机。
13. 我承认不管手术的结果如何，我都应该支付毛囊黑素细胞干细胞移植手术的费用，我知道为手术支付手术费用是因为手术本身，而不是因为预期的结果。

患者签名 郭文鹏　　　日期 2020.7.3

见证人签名 ________　　　日期 ________

可能发生的并发症

*出血（低于 1%） *感染（低于 1%）*暂时性的头皮麻木 *移植物周围偶发疤痕 *晕厥（低于 1%）与晕厥发作 *偶尔向内生长的头发形成囊肿（低于 5%）*供区疤痕偶见，低于（5%）

吸烟的患者更容易发生伤口愈合延缓和毛囊黑素细胞干细胞生长不佳。建议在术后 2-3 周不要吸烟。

罕见的并发症（仅列出部分）

*瘢痕疙瘩 *供区头发全部脱落 *头皮永久性麻木 *过敏反应或药物相关的反应

我已经阅读并且知道了上述列出的可能发生的并发症。我愿意承担手术过程中可能发生的并发症和风险。

患者签名 郭文鹏　　　日期 2020.7.3

见证人签名 ________　　　日期 ________

手术麻醉的知情同意：

1. 任何一种麻醉都存在风险，因此并不能确保每一项操作或治疗的后果。然而，麻醉过程中发生不可预测的严重反应非常罕见，包括：很少发生的感染、出血、药物反应、血栓、感觉缺失、丧失肢体功能、麻痹、中风、脑损伤、心脏病发作和死亡。
2. 我知道自己手术过程中所使用的麻醉方法（以下列出），选择何种麻醉方法是由许多因素决定的，如患者的身体状况、医师的偏爱以及患者自己的要求。
3. 神经阻滞非常有效，而且通常比较安全。但是在非常少的患者中，也报道可以发生神经损伤，发生率大约为 1:30000，而且绝大多数患者可以自行恢复，不需要进行治疗。

   使用的麻醉：**不使用镇静药进行主要或次要的神经阻滞。**

   预期的结果：暂时性的感觉丧失和（或）累及特定的部位。

   方法：在神经周围注射药物。

   风险：包括（但并不局限于）感染、惊厥、虚弱、持续性麻木、后遗痛，血管损伤或神经损伤。

   我知道并同意上述列出的麻醉，我同意由 刘景卫 医师和（或）助手进行麻醉。我也同意，必要时可以更换合适的麻醉方法。

我确定我已经阅读了上述内容，或者医师已经向我阅读了上述内容，我知道麻醉的风险，预期效果及其他可以选择的麻醉方法。我有足够的时间来进行提问和作出决定。

以上条款已向患者或亲属交代清楚并表示理解和同意，签名为证。

专科情况：	
本人对上述情况已表示完全理解，同意手术<br>患者或家属签名： 郭义鹏<br>与患者的关系<br>2020 年 7 月 3 日	谈话医生签名：<br>手术负责医生签名： 刘景卫<br>2020 年 7 月 3 日
